# Varying Selection Pressure for a Na^+^ Sensing Site in Epithelial Na^+^ Channel Subunits Reflect Divergent Roles in Na^+^ Homeostasis

**DOI:** 10.1093/molbev/msae162

**Published:** 2024-08-05

**Authors:** Xue-Ping Wang, Priyanka Srinivasan, Mustapha El Hamdaoui, Brandon M Blobner, Rafael Grytz, Ossama B Kashlan

**Affiliations:** Renal-Electrolyte Division, Department of Medicine, University of Pittsburgh, Pittsburgh, PA, USA; Renal-Electrolyte Division, Department of Medicine, University of Pittsburgh, Pittsburgh, PA, USA; Department of Ophthalmology and Visual Sciences, University of Alabama at Birmingham, Birmingham, AL, USA; Department of Bioinformatics, BlueSphere Bio, Pittsburgh, PA, USA; Department of Ophthalmology and Visual Sciences, University of Alabama at Birmingham, Birmingham, AL, USA; Renal-Electrolyte Division, Department of Medicine, University of Pittsburgh, Pittsburgh, PA, USA; Department of Computational and Systems Biology, University of Pittsburgh, Pittsburgh, PA, USA

**Keywords:** purifying selection, allostery, sodium homeostasis, paralog divergence

## Abstract

The epithelial Na^+^ channel (ENaC) emerged early in vertebrates and has played a role in Na^+^ and fluid homeostasis throughout vertebrate evolution. We previously showed that proteolytic activation of the channel evolved at the water-to-land transition of vertebrates. Sensitivity to extracellular Na^+^, known as Na^+^ self-inhibition, reduces ENaC function when Na^+^ concentrations are high and is a distinctive feature of the channel. A fourth ENaC subunit, δ, emerged in jawed fishes from an α subunit gene duplication. Here, we analyzed 849 α and δ subunit sequences and found that a key Asp in a postulated Na^+^ binding site was nearly always present in the α subunit, but frequently lost in the δ subunit (e.g. human). Analysis of site evolution and codon substitution rates provide evidence that the ancestral α subunit had the site and that purifying selection for the site relaxed in the δ subunit after its divergence from the α subunit, coinciding with a loss of δ subunit expression in renal tissues. We also show that the proposed Na^+^ binding site in the α subunit is a bona fide site by conferring novel function to channels comprising human δ subunits. Together, our findings provide evidence that ENaC Na^+^ self-inhibition improves fitness through its role in Na^+^ homeostasis in vertebrates.

## Introduction

Epithelial Na^+^ channels (ENaC) appeared in early vertebrates and play important roles in Na^+^ sensing and regulating extracellular fluids. The channel is typically expressed on cell surfaces, where its large extracellular domain can sense and respond to the extracellular environment (Kashlan et al. 2024). Accordingly, adaptive changes in ENaC function have paralleled vertebrate evolution. For example, cleavage sites that facilitate constitutive activation of the channel coevolved with the terrestrial migration of vertebrates, likely selected for by the increased threat of desiccation that terrestrial habitats pose (Kashlan, Balchak, et al. 2018; Wichmann et al. 2019; Wang, Balchak, et al. 2022). ENaC also directly senses Na^+^ and other small molecules present in extracellular fluids (Kashlan et al. 2024).

**Fig. 1.**
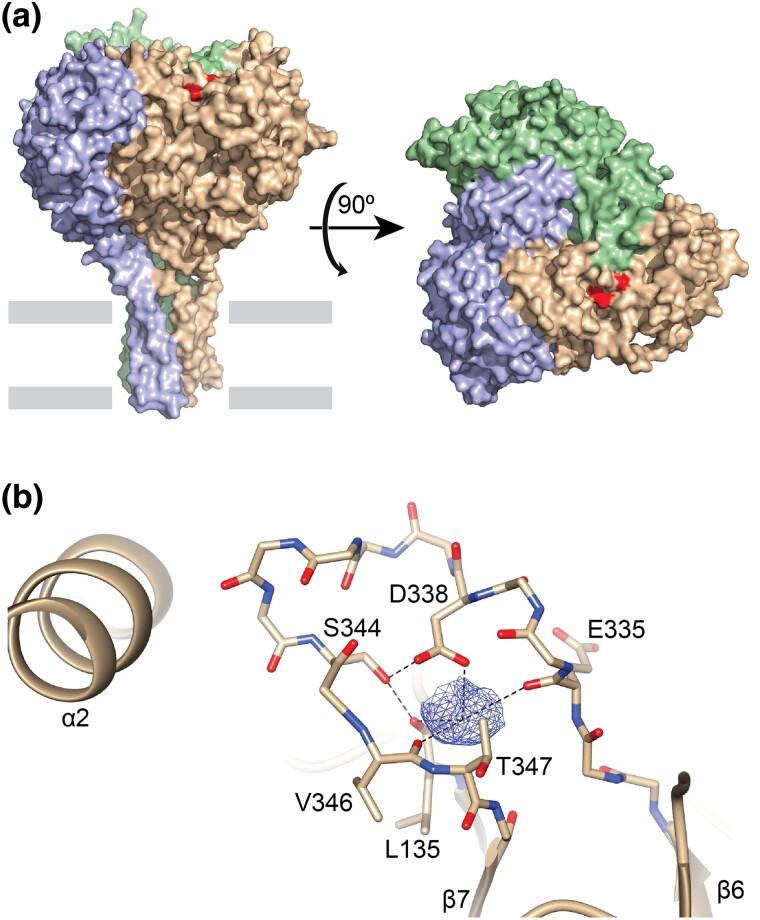
Cation-binding site in the β6 to β7 loop of the α subunit. a) Surface model of human ENaC trimer (pdb code: 6BQN) made of α, β, and γ subunits, front-right, rear, and front-left, respectively. Residues implicated in cation binding are highlighted in the α subunit, near the α/β subunit interface. Bars indicate approximate location of the membrane. b) Cartoon and sticks representation of cation-binding pocket in the α subunit (pdb code: 6WTH). Carboxylate, hydroxide, and carbonyl groups from residues in the β6 to β7 loop and L135 contribute to the binding site. Dashed lines illustrate distances shorter than 3.5 Å.

The inhibition by extracellular Na^+^, known as Na^+^ self-inhibition, was first observed in frog skin when rapid increases in extracellular Na^+^ initially increased currents as expected for a Na^+^ selective channel, but then reduced currents over a few seconds (Fuchs et al. 1977). Evidence supports Na^+^ binding at one or more extracellular effector sites that drive down the channel's open probability (*P*_o_) (Sheng et al. 2006; Kashlan et al. 2015). In humans and mice, binding likely occurs at a site in the α subunit (SCNN1A) centered around human Asp-338 (mouse Asp-365) in the β6 to β7 loop of the β-ball domain (Fig. 1). This site was first identified in mouse ENaC by examining the cation specificity of inhibition, effects on H^+^-dependent activation, and crosslinking experiments connecting local conformational changes to gating (Kashlan et al. 2015). Even conservative mutations at this site (Asn or Glu) weakened Na^+^ self-inhibition. Wichmann et al. (2019) confirmed key observations in both the α and δ (SCNN1D) subunits from *Xenopus laevis*. Noreng et al. (2020) observed electron density near Asp-338 in the human α subunit consistent with a bound cation and speculated that it was a Na^+^ ion.

ENaCs are heterotrimers composed from four paralogous subunits: α (SCNN1A), β (SCNN1B), γ (SCNN1G), and δ (SCNN1D). *SCNN1A*, *SCNN1B*, and *SCNN1G* arose from two gene duplication events early in vertebrates and appear in each of the vertebrate classes (Studer et al. 2011). However, these genes disappeared in ray-finned fishes after the divergence of Polypteriformes (Evans et al. 2005; Wang, Balchak, et al. 2022). *SCNN1D* emerged from an *SCNN1A* duplication in a jawed-fish ancestor (Wang, Balchak, et al. 2022), and has been found in the coelacanth and tetrapods. *SCNN1D* was subsequently lost in a subset of rodents including mice and rats (Giraldez et al. 2012; Gettings et al. 2021), which has led to a poor understanding of its physiological role. ENaC α and δ subunits are interchangeable in assembled channels and confer distinct functional properties. For example, human αβγ ENaC shows robust Na^+^ self-inhibition and is activated by proteases, whereas human δβγ ENaC is less sensitive to both Na^+^ and proteases (Ji et al. 2006; Haerteis et al. 2009). Other trimeric assemblies are also functional and likely occur when subunits are limiting (Canessa et al. 1994; Waldmann et al. 1995; Haerteis et al. 2009; Wang, Tomilin, et al. 2022), but their functional properties have received little attention and their roles in physiology are poorly understood (Weisz and Johnson 2003; Kashlan et al. 2024). In contrast to the human α subunit, the human δ subunit lacks an Asp at the analogous position associated with Na^+^ self-inhibition, as do δ subunits from several other mammalian species (Wichmann et al. 2019). These include the guinea pig, and accordingly, Na^+^ self-inhibition is weaker for *Cavia porcellus* ENaCs comprising SCNN1D when compared with SCNN1A (Gettings et al. 2021). Intriguingly, both the α and δ subunits from *X. laevis*, rooted close to the α/δ subunit divergence point, retain the analogous Asp residue and demonstrate Na^+^ self-inhibition (Wichmann et al. 2019).

Based on these data, we hypothesized that residues involved in Na^+^ binding were present before the divergence of the α and δ subunits, and then selectively lost in the δ subunit after the divergence. Consequently, we hypothesized that a species closely related to humans retains the site and that we could resurrect a Na^+^ self-inhibition site in the human δ ENaC subunit. We determined the phylogenetic relationship of α and δ subunits and analyzed the appearance of key residues at the proposed Na^+^ self-inhibition site. The central Asp demonstrated markedly different inheritance patterns after the divergence of the α and δ subunits. Analysis of the nonsynonymous to synonymous substitution rate ratios (dN/dS) of the coding sequences provides evidence for purifying selection of residues comprising the site in the α subunit, but relaxed selection pressure for the analogous residues in the δ subunit. We identified a close human relative in the order *Scandentia* that retained the homologous Asp in its δ subunit and shows Na^+^ self-inhibition that depends on the same. After identifying site differences with human δ, we were able to confer Na^+^ self-inhibition to human δβγ channels through selective mutagenesis. In contrast to consistently observing renal expression of the α subunit, we did not detect ENaC δ subunit expression in kidneys from the treeshrew, opossum, green anole lizard, or swan goose, suggesting renal expression of the δ subunit is not conserved. Taken together, our data unambiguously define a Na^+^ self-inhibition site and provide evidence that Na^+^ self-inhibition improves fitness through its function in the kidney.

## Results

### Conservation of Asp Associated with Na^+^ sensing

**Fig. 2.**
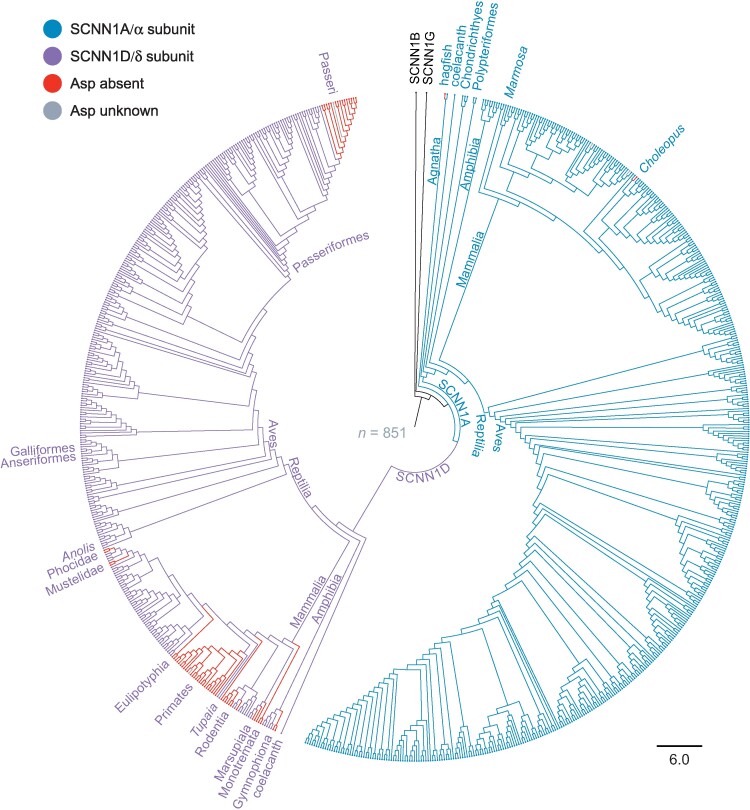
Phylogenetic analysis of ENaC α and δ subunits. Circular phylogram representation of maximum-likelihood tree calculated from 849 SCNN1A and SCNN1D sequences, along with human SCNN1B and SCNN1G subunits. The tree was rooted using human SCNN1B. Branches are colored according to the residue aligning with human αAsp-338: Asp in α subunits is blue, Asp in δ subunits is purple, non-Asp residues are red, and unknown residues are gray. Select internal branch points and taxons are highlighted. Scale bar indicates the number of substitutions per site.

To examine the evolution of human αD338 and other residues implicated in Na^+^ binding for Na^+^ self-inhibition, we determined the phylogenetic relationship of 849 SCNN1A and SCNN1D sequences (Fig. 2 and supplementary table S1, Supplementary Material online). We rooted the tree using human SCNN1B as the outgroup. Our tree exhibits the expected phylogenetic relationships between the subunits based on two gene duplication events before the emergence of Agnatha (Wichmann and Althaus 2020; Wang, Balchak, et al. 2022), an *SCNN1A* duplication event in a jawed fish giving rise to *SCNN1D*, and speciation thereafter. One key exception is the placement of *Latimeria chalumnae* (coelacanth) SCNN1A basal to SCNN1A from Chondrichthyes and Polypteriformes, reflecting weak branch support values previously observed for SCNN1A and SCNN1D shortly after their divergence (Wang, Balchak, et al. 2022). When we examined the site aligning with human αD338 in SCNN1A, we found an Asp in every sequence except in *Eptatretus burgeri* (Asn) (Wichmann et al. 2019) before the emergence of SCNN1D, and in *Choloepus didactylus* (Gly) after the divergence of SCNN1A and SCNN1D. In SCNN1D, 59 species had a residue other than Asp at the same position (Fig. 2 and supplementary table S2, Supplementary Material online). These include the Gymnophiona order of amphibians (Asn), all primates (Ala or Pro), several other mammalian groups (Lys, Asn, Ser, His, Val, Tyr, or Gly), and several members of the Passeri suborder of perching birds (Ala or Glu). We also identified *Tupaia belangeri* (treeshrew) in the order Scandentia and *Galeopterus variegatus* (flying lemur) in the order Dermoptera as the closest human relatives with a δ subunit bearing an Asp in the same position in the alignment.

### Treeshrew δ Exhibits Na^+^ Self-inhibition

**Fig. 3.**
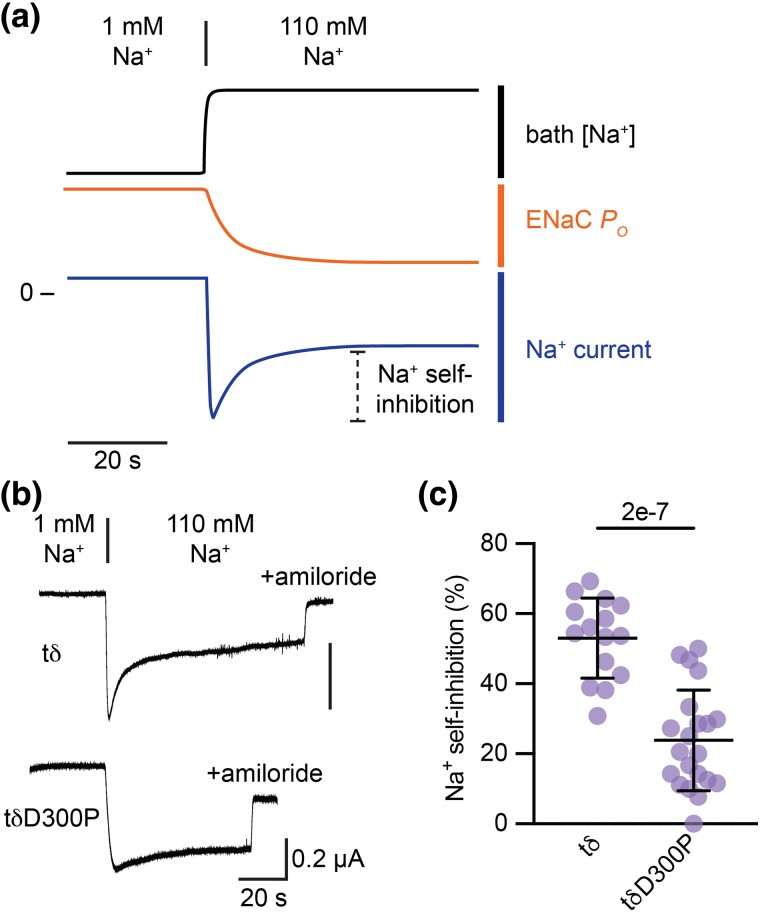
Conserved Asp in treeshrew δ ENaC subunit affects Na^+^ self-inhibition. a) Schematic of voltage-clamp experiment to measure Na^+^ self-inhibition. With oocytes mounted in a 20 µL chamber perfused at 5 mL/min and clamped at −100 mV, bath [Na^+^] is rapidly increased from 1 mM Na^+^ to 110 mM Na^+^. This rapidly increases the driving force for Na^+^ entry and results in an inward current peak shortly thereafter. Simultaneously, extracellular Na^+^ binds ENaC and decreases *P*_O_ with a time constant of ∼2–3 s. Declining currents from the peak reflect decreases in *P*_O_. b) Representative recordings of *Xenopus* oocytes injected with the tδ subunit indicated and complementary hβ and hγ ENaC subunits. Amiloride (10 µM) was added in 110 mM Na^+^ buffer to determine ENaC-specific currents. c) Na^+^ self-inhibition was assessed as the reduction of current from the peak to steady state 1 min after increasing bath [Na^+^] relative to the total amiloride-sensitive current measured from the peak to the value with amiloride. Groups were compared by Student's *t*-test.

We hypothesized that the treeshrew δ subunit (tδ) has an intact Na^+^ binding site. We therefore synthesized tδ and expressed it along with wild-type human β (hβ) and γ (hγ) ENaC subunits in *X. laevis* oocytes, and then assessed its function using two-electrode voltage-clamp current recordings. While clamping oocytes at −100 mV, we rapidly increased bath [Na^+^] from 1 to 110 mM Na^+^ in a 20 µL chamber perfused at 5 mL/min. This maneuver takes advantage of ENaC's slow gating kinetics (∼0.2 opening events/s and 0.8 closing events/s [Anantharam et al. 2006]) relative to bath exchange rates (∼4 bath volumes/s) to measure Na^+^ self-inhibition (Fig. 3a) (Chraibi and Horisberger 2002). With 1 mM Na^+^ in the bath, ENaC's *P*_O_ is relatively high. This results in an inward current peak when the driving force for inward currents is suddenly increased by an increase in bath [Na^+^]. Na^+^ then binds an extracellular effector site that drives down ENaC's *P*_O_, rate limited by ENaC's gating kinetics (Chraibi and Horisberger 2002). Oocytes expressing tδ subunits exhibited robust Na^+^ self-inhibition (Fig. 3b and c). To test whether the conserved Asp in tδ (tδD300) affects Na^+^ self-inhibition, we mutated it to its human δ equivalent, Pro. Mutation decreased the Na^+^ self-inhibition response, but did not abolish it (*P* = 0.007 for peak vs. steady-state currents by paired Student's *t*-test). These data are consistent with a role for tδD300 in Na^+^ binding, but also suggest residual Na^+^ binding in the tδD300P mutant.

### Selection Pressure Was Lost for Functional Site Residues in ENaC's δ Subunit

The pattern of Asp residues in our tree at the position aligning with human αD338 in extant species raises several questions. Do trait loss rates differ between the α and δ subunits? Did ancestral subunits have an Asp at the site? What modes of selection are operating on residues implicated in Na^+^ binding?

**Fig. 4.**
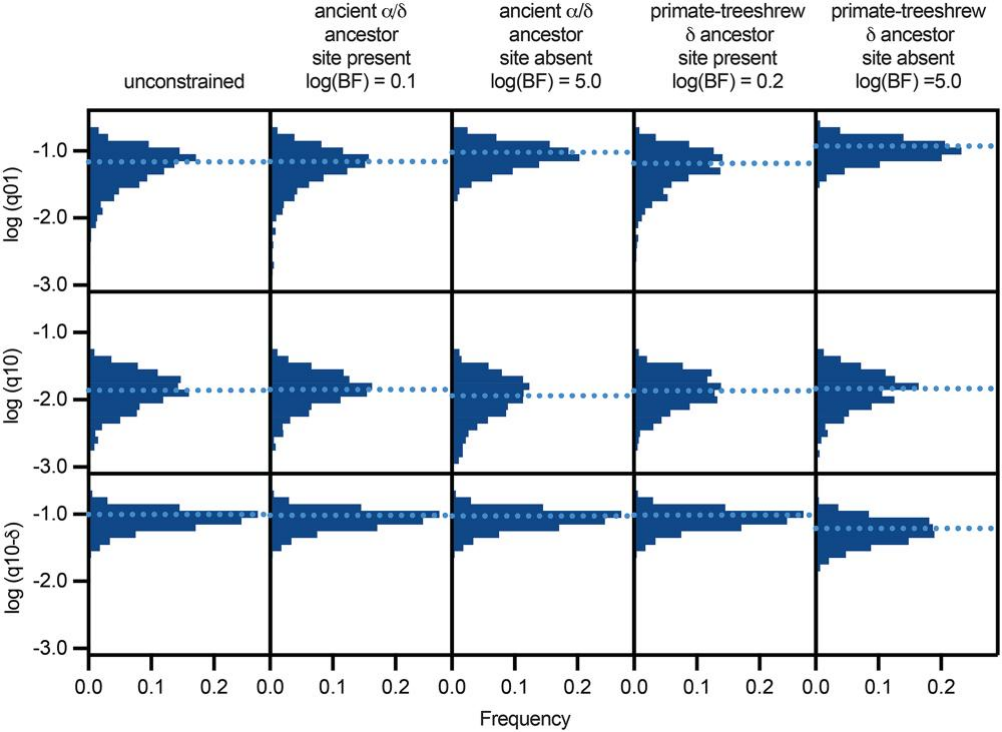
Fitted model parameters for Asp trait evolution. Trait evolution across the phylogenetic tree was determined using BayesTraits. MCMC runs were performed to determine the effect on model fits of fixing an ancient α/δ ancestor (the most recent common ancestor to sea lamprey α, coelacanth α, and coelacanth δ) or the primate-tree shrew δ subunit ancestor (the most recent common ancestor to human δ, treeshrew δ, and flying lemur δ) to either have or not have the Asp trait. Histograms of log-transformed rates from every 1,000th iteration after the first 10,000 iterations are shown. Median values for each distribution are indicated with light dotted lines. Log Bayes Factors [log(BF)] are shown for each ancestrally constrained model when compared with the unconstrained model.

To test for changes in trait loss rates after subunit divergence, we compared two nested models of site evolution over the full phylogenetic tree using BayesTraits and maximum-likelihood methods (Pagel et al. 2004). We assigned each sequence as either having or lacking an Asp according to Fig. 2. For SCNN1B, SCNN1G, and the four sequences in our tree lacking sequence information for the site (gray in Fig. 2), we assigned “unknown.” The independent model, serving as the null hypothesis, contained two parameters: a site gain rate (q01) and a site loss rate (q10) common to both subunits. The dependent model added one free parameter by having an independent loss rate for SCNN1D vertebrate classes (q10–δ) where the site was lost (amphibians, mammals, and birds). A likelihood ratio test comparing the models provided strong evidence favoring the dependent model (*P* = 0.007). In an MCMC run of the favored model (Fig. 4, unconstrained), median values for the q01:q10 ratio were 4.4, favoring site retention, compared with 0.72 for the q01:q10–δ ratio, slightly favoring site loss.

To resolve whether the ancient α/δ ancestral subunit had an Asp at the site, we compared fits of the dependent model to the tree using MCMC methods while fixing the state of the site (present or absent) in the most recent common ancestor to the Agnathan sea lamprey α, coelacanth α, and coelacanth δ subunits. Constraining the ancient ancestor to lack the Asp increased the median q01 value by 35% and decreased the median q10 value by 16% compared with the unconstrained model (Fig. 4). In contrast, fixing this early ancestor to have an Asp at the site had little effect on model rates, with the medians for all rate distributions within 3% of the unconstrained model. We compared fits by converting marginal likelihoods from each MCMC run to log Bayes Factors, where values <2 provide weak evidence, >2 provide positive evidence, and >5 provide strong evidence of a preference between models. Fixing the ancient ancestor to have the Asp gave a log Bayes Factor of 0.1 vs. the unconstrained model, while fixing the ancient ancestor to lack the Asp gave a log Bayes Factor of 5.0 vs. the unconstrained model. Comparing the constrained models with each other gave a log Bayes Factor of 4.9, favoring the presence of an Asp in the ancestral node. These data provide evidence that the ancestor basal to both SCNN1A and SCNN1D likely had an Asp at the site. We performed a similar analysis to determine whether the most recent common ancestor to human δ and treeshrew δ had an Asp at the site (Fig. 4). Fixing the primate-treeshrew δ ancestor to lack the site shifted both q01 (64% faster) and q10–δ (36% slower), compared with the unconstrained model, while fixing this ancestor to have the site had little effect on model parameters (1% to 5%). Accordingly, log Bayes Factors comparisons (Fig. 4) favor the presence of an Asp in this ancestor. These data provide evidence that this more recent ancestor had the site and that it was lost on the primate lineage after their divergence from Scandentia 73 to 100 million years ago (Zhou et al. 2015).

To determine whether a change in selection pressure after the divergence of the α and δ subunits explains the difference in site retention rates, we compared the nonsynonymous substitution rate (dN) and synonymous substitution rate (dS) for the sequences encoding key residues implicated in Na^+^ self-inhibition (Fig. 1). Values of dN/dS close to 1 are consistent with neutral drift, values >1 indicate diversifying selection, and values <1 suggest purifying selection (Yang et al. 2000). We used HYPHY to measure and compare dN and dS for the key sites in each subunit (Kosakovsky Pond et al. 2020). Using HYPHY's FEL module (Kosakovsky Pond and Frost 2005), dN/dS was <1 for all selected sites, except for the δ subunit site aligning with hαV346 (Table 1). This is consistent with purifying selection at each of these sites. As the values for α appeared smaller than for δ, we then explicitly compared them using HYPHY's Contrast-FEL module (Kosakovsky Pond et al. 2021). Values were smaller in the α subunit for sites aligning with hαL135, hαD338, hαS344, and hαV346. Strikingly, there was a 25-fold difference in dN/dS for the site aligning with hαD338. These data indicate that purifying selection pressure relaxed for several of the residues implicated in Na^+^ binding after the δ subunit diverged from the α subunit.

**Table 1:** Analysis of codon substitution rates

	α subunit		δ subunit		
Site (hα)	dN/dS	*P*(dN < dS)	dN/dS	*P*(dN < dS)	*P*(α < δ)
L135	0.015	<0.001	0.10	<0.001	0.03
E335	0.062	<0.001	0.15	<0.001	0.1
D338	0.020	<0.001	0.50	0.03	<0.001
S344	0	<0.001	0.17	<0.001	0.002
V346	0.12	0.004	0.74	0.39	<0.001
T347	0.024	<0.001	0.060	<0.001	0.2

Analysis of dN and dS for residues implicated in Na^+^ binding (see Fig. 1). *P*(dN < dS) was determined using FEL. The probability that dN for the α subunit is less than dN for the δ subunit, assuming a common dS [*P*(α < δ)], was determined using Contrast-FEL.

### Weakened Selection Pressure Coincides with Loss of Renal δ ENaC Subunit Expression

**Fig. 5.**
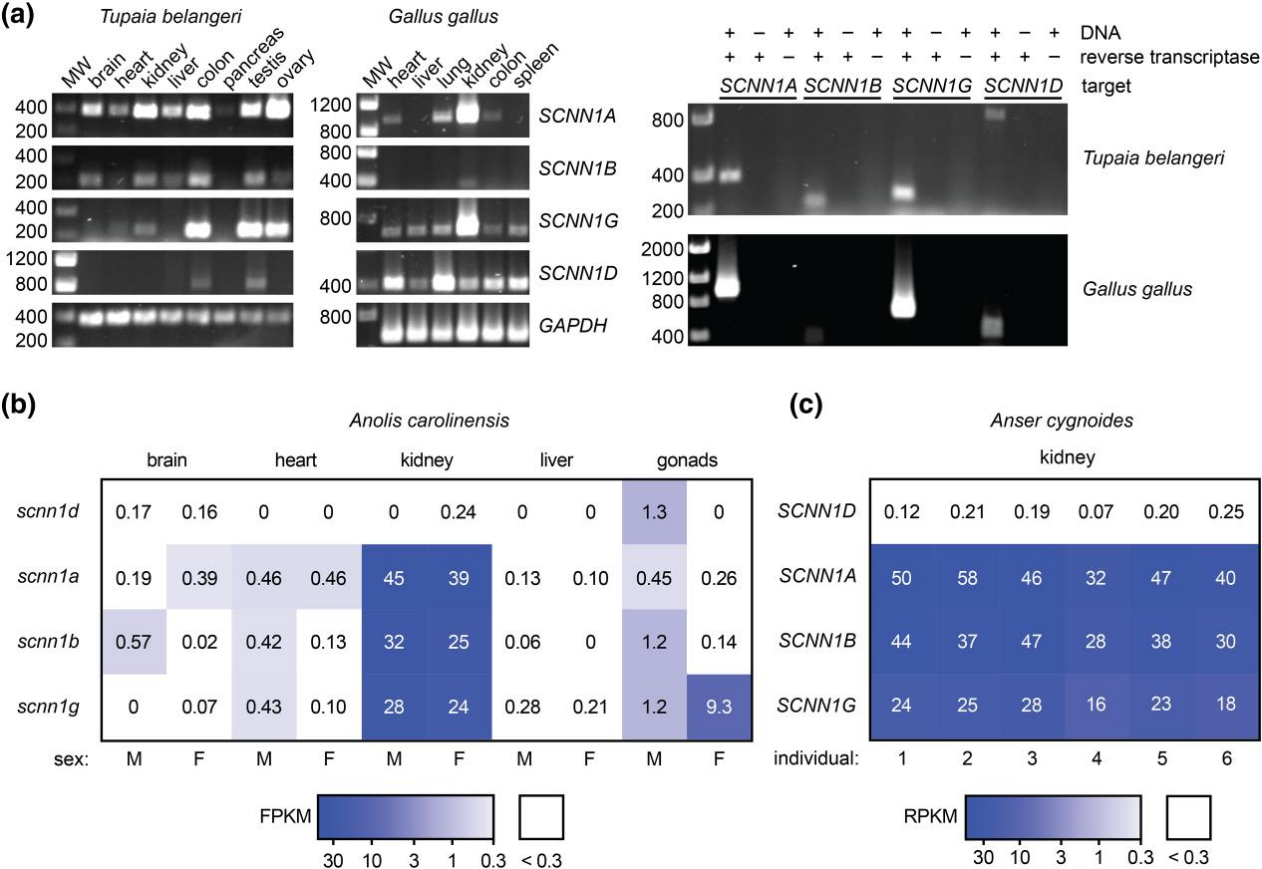
Tissue expression of ENaC subunit transcripts in selected species. *a, Left*) Transcript expression in *T. belangeri* and *G. gallus*. Tissue homogenates were used to generate cDNA libraries. PCR reactions were performed using primer pairs indicated in supplementary table S3, Supplementary Material online. *Right*) The kidney was used for negative controls, except for *SCNN1D* from *T. belangeri*, where the testis was used. *b*) RNA-seq data for *A. carolinensis* are from GEO (ncbi.nlm.nih.gov/geo) accession GSE97367 (Marin et al. 2017). Expression data units are fragments per kilobase per million mapped fragments (FPKM), where values <0.3 were considered background (Hart et al. 2013). *c*) RNA-seq data for *A. cygnoides* are from GEO accession GSE144723. Expression data units are reads per kilobase per million mapped reads (RPKM), where values <0.3 were considered background (Ramskold et al. 2009). Orthologous genes were identified using genenames.org.

We reasoned that ENaC Na^+^ self-inhibition is important for renal function since Na^+^ concentrations can vary widely in the distal nephron, e.g. 10 to 220 mM Na^+^ in tubular fluid from the rat's distal nephron (Malnic et al. 1966). ENaC Na^+^ self-inhibition is well tuned to this range, with apparent affinity weaker than 100 mM, and inhibitory effects evident at ∼10 mM Na^+^ (Chraibi and Horisberger 2002; Sheng et al. 2006). Kidney lysates from *X. laevis* frogs express both ENaC α and δ subunits (Wichmann et al. 2018; Wang, Balchak, et al. 2022). ENaC δ subunit expression in human kidneys appears to be low or absent (Menon et al. 2020). In treeshrew tissue lysates, we observed bands for the α, β, and γ subunit transcripts in the kidney, but no signal for the δ subunit transcript (Fig. 5a). Like human (Waldmann et al. 1995), we readily detected δ subunit transcript expression in treeshrew testis. Unlike human, we did not detect δ subunit transcripts in treeshrew brain, pancreas, or ovary tissues. We analyzed kidney tissue from *Marmosa mexicana*, a marsupial rooted close to the base of the mammals. As we were unable to amplify ENaC transcripts using primers based on related opossum species, we sequenced the transcripts in our kidney tissue sample. We identified transcripts for the α, β and γ ENaC subunits, but not the δ subunit, in *M. mexicana* kidney tissue (supplementary file S5, Supplementary Material online; NCBI BioProject PRJNA1052681).

We also investigated ENaC subunit expression in *Anolis carolinensis* (green anole lizard)*, Gallus gallus* (chicken), and *Anser cygnoides* (swan goose). In deposited RNA-seq data from *A. carolinensis* (GSE97367 [Marin et al. 2017]), we found robust expression of *scnn1a, scnn1b,* and *scnn1 g* in kidney tissues (Fig. 5b). In contrast, fragment reads for *scnn1d* in the kidney were absent in males and below the background threshold in females. In the dataset, *scnn1d* expression exceeded background levels only in the testis. In tissues from *G. gallus* (Fig. 5a), we detected transcripts for all four ENaC subunits in kidney lysates. In contrast to the pattern of expression in mammals and in the anole lizard, *SCNN1D* expression was widespread. In deposited RNA-seq data from *A. cygnoides* kidney tissues (GSE144723) from six healthy goslings, we found a robust signal for *SCNN1A*, *SCNN1B*, and SCNN1G (Fig. 5c). However, like in the lizard, the expression of *SCNN1D* was below the background threshold in each of the goslings. As δ subunit transcript expression has been observed in kidneys from the frog and chicken, but not the human, treeshrew, opossum, anole lizard or swan goose, our data indicate that renal δ subunit expression is poorly conserved, in contrast to renal expression of the α, β, and γ subunit transcripts.

### Na^+^ Binding Site Resurrection in Human δ ENaC

**Fig. 6.**
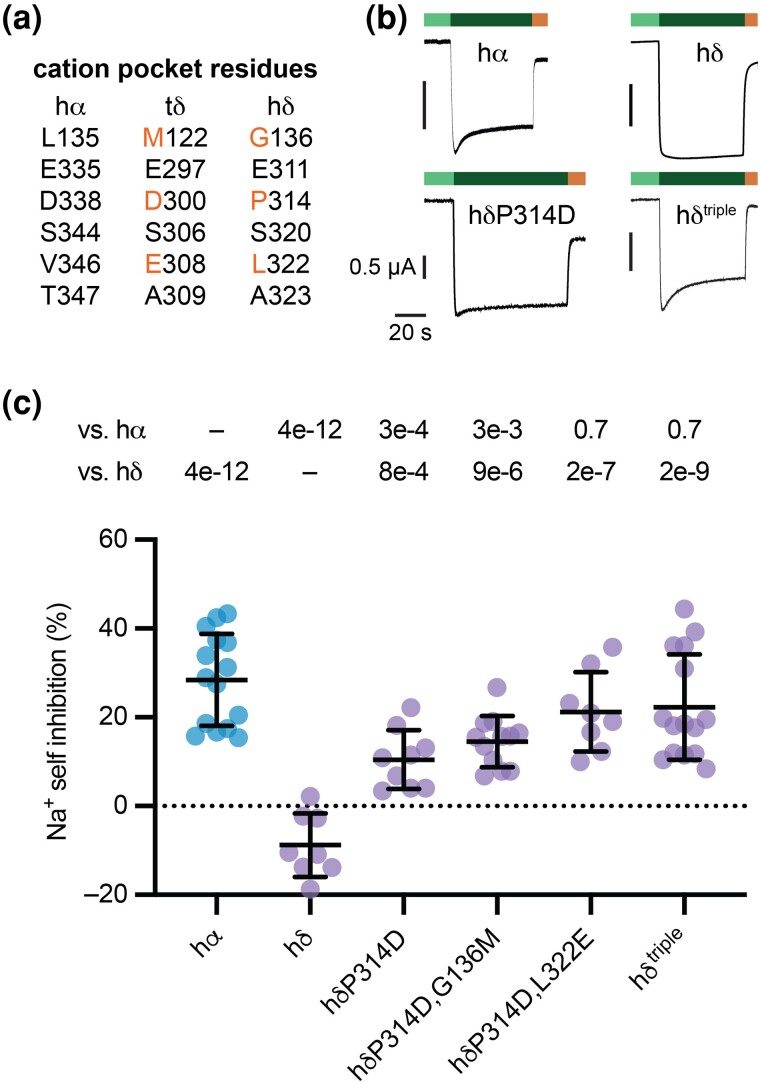
Na^+^ binding site resurrection in the δ subunit of human ENaC. a) Key resides involved in the cation-binding pocket in hα, along with their tδ and hδ equivalents. b) Representative recordings of *Xenopus* oocytes injected with the hα or hδ subunit indicated and complementary hβ and hγ ENaC subunits. Bath conditions are indicated using colored bars: 1 mM Na^+^ (light green), 110 mM Na^+^ (dark green), and 10 µM amiloride in 110 mM Na^+^ buffer (orange). c) Na^+^ self-inhibition was assessed as the relative loss of amiloride-sensitive current from the peak to steady state, as above, and groups were compared using one-way ANOVA and Šidák’s multiple comparisons test.

Given the likely presence of Na^+^ self-inhibition in the most recent common ancestor to tδ and hδ, and its loss in primates thereafter, we hypothesized that we could reconstruct a Na^+^ binding site in hδ and confer Na^+^ self-inhibition to channels comprising hδ subunits. We identified key differences in the cation-binding pocket between the hδ and tδ subunits at the site, and mutated hδ accordingly (Fig. 6a). Mutating hδP314 to Asp produced a discernable current peak after increasing extracellular [Na^+^] (Fig. 6b and c). Additionally mutating hδG136 to Met and/or hδL322 to Glu further increased Na^+^ self-inhibition, so that responses for channels comprising double and triple hδ subunit mutants were similar to channels comprising the hα subunit. Each hδ mutant increased the response to extracellular Na^+^ compared with wild-type hδ. These data conferring novel function to hδ provide evidence that hαD338 and neighboring residues (Fig. 1) constitute a bona fide inhibitory Na^+^ effector site.

## Discussion

**Fig. 7.**
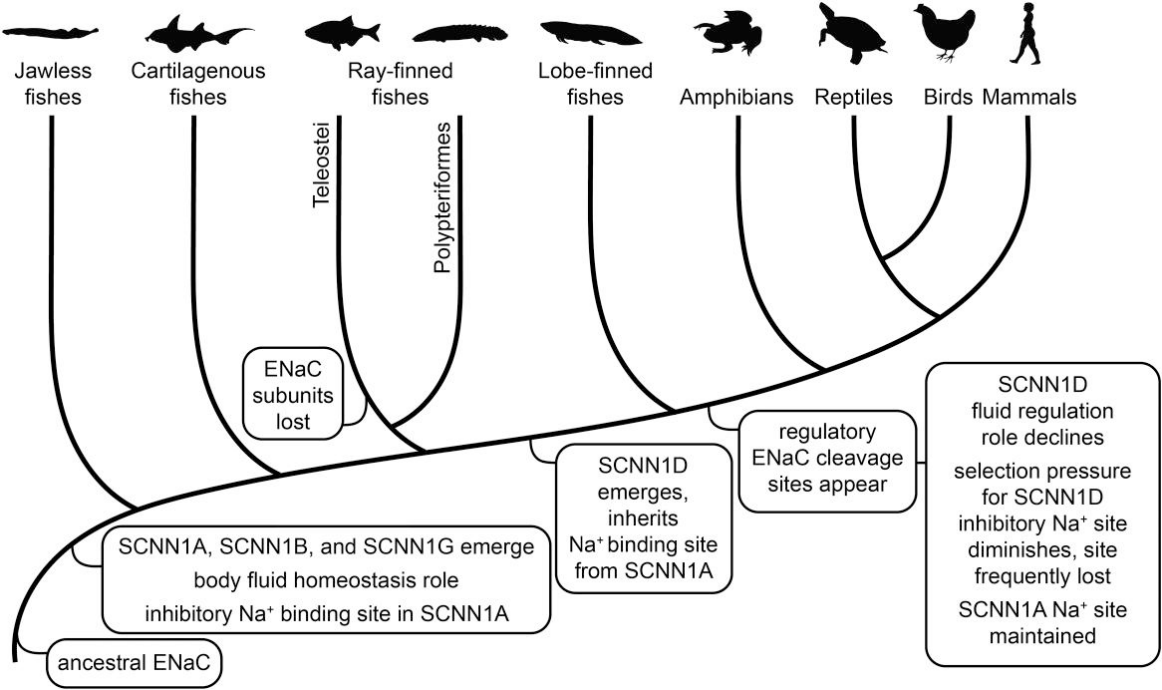
Evolution of ENaC subunits and regulatory motifs. Distinct ENaC α, β, and γ subunits appeared early in vertebrate evolution, with expression consistently found in organs responsible for maintaining Na^+^ homeostasis. The ancestral channel also likely had an inhibitory Na^+^ binding site in its α subunit that made the channel sensitive to extracellular Na^+^. This site was present well before motifs for proteolytic activation of the channel appeared during the vertebrate land invasion. In a jawed fish ancestor, the δ subunit emerged from a α subunit gene duplication event, inheriting its inhibitory Na^+^ binding site. Afterwards, selection pressure to maintain the Na^+^ binding site relaxed in the δ subunit, leading to its loss in several species. This loss coincides with a loss of δ subunit expression in kidney tissues from numerous species, in contrast to conserved renal expression of the α subunit. These observations suggest that ENaC's role in total body Na^+^ and fluid homeostasis underlie the selection pressure that maintained the Na^+^ binding site in the α subunit. Figure adapted from the previous work (Wang, Balchak, et al. 2022). Animal silhouettes courtesy of PhyloPic (http://www.phylopic.org).

ENaC subunits emerged early in vertebrates, with α, β, and γ subunits present in jawless fishes (Fig. 7). Our findings provide evidence that this ancestral ENaC was inhibited by Na^+^ binding to an extracellular site in the channel's α subunit and that this functional property improved fitness throughout vertebrate evolution. Although first observed five decades ago (Fuchs et al. 1977), the physiological relevance of ENaC Na^+^ self-inhibition remains poorly understood (Kashlan et al. 2024). Its relevance has been linked to ENaC's sensitivity to other regulatory factors, e.g. proteases, activation through which has been proposed to depend on Na^+^ self-inhibition (Sheng et al. 2006; Noreng et al. 2020). However, the apparent functional link can also be explained through indirect mechanisms (Kashlan et al. 2010; Passero et al. 2010). Furthermore, the evolution of ENaC functional sites does not support this (Fig. 7). Purifying selection maintained an inhibitory Na^+^ binding site in the α subunit throughout vertebrate evolution, both before and after sites for proteolytic activation emerged in the α and γ subunits with the migration of vertebrates to terrestrial habitats (Kashlan, Balchak, et al. 2018; Wichmann et al. 2019; Wang, Balchak, et al. 2022). Unlike proteolytic activation, proton-dependent activation is directly connected to Na^+^ self-inhibition, since protons activate the channel by directly competing with Na^+^ for the key Asp (Collier and Snyder 2009; Kashlan et al. 2015; Wichmann et al. 2019). However, ENaC pH sensitivity varies widely between species and protonation of the Asp involved in Na^+^ binding appears to be only part of the mechanism (Collier et al. 2012; Wichmann et al. 2019). We, therefore, conclude that ENaC sensitivity to extracellular Na^+^ per se improves fitness.

We propose that the loss of renal expression caused the relaxation of purifying selection for Na^+^ binding residues in the δ subunit. After the divergence of the α and δ subunits, δ subunit expression in renal tissues appears to have been lost on some branches. Renal δ subunit transcript expression has been detected in *X. laevis* frogs and chicken; however, we and others detected little or no δ subunit transcript expression in human, treeshrew, opossum, anole lizard, or swan goose kidney tissues (Fig. 5) (Giraldez et al. 2007; Wichmann et al. 2018; Menon et al. 2020; Wang, Balchak, et al. 2022). Conversely, the α subunit transcript is expressed in the kidneys of each of these species (Menon et al. 2020; Wang, Balchak, et al. 2022), and in the kidneys of the ropefish and two species of lungfish, in which ENaC δ subunits have not been detected (Uchiyama et al. 2012, 2015; Wang, Balchak, et al. 2022). An important limitation of these data is the reliance on transcript detection rather than analysis of noncoding regulatory elements (Kelley 2020; Avsec et al. 2021). Nevertheless, the notion that ENaC Na^+^ self-inhibition improves renal function is attractive. Na^+^ concentrations in the distal renal tubule are highly variable, unlike many other sites that express ENaC where Na^+^ concentrations are constitutively high, such as in lung fluid and blood. Tonic inhibition through Na^+^ self-inhibition could increase fitness under these conditions, though other modest loss-of-function mutations could produce a similar effect. On the other hand, the apparent affinity for Na^+^ binding to ENaC, while weak, is well matched to the distal renal tubule Na^+^ concentrations measured in rats (Malnic et al. 1966). The net effect of Na^+^ self-inhibition is to maximize Na^+^ transport when extracellular Na^+^ concentrations are low, and dampen Na^+^ transport when extracellular Na^+^ concentrations are high. This effectively sharpens the channel's Na^+^ saturation curve so that Na^+^ transport rates rise steeply as Na^+^ concentrations increase to 20 mM, but then plateau at higher concentrations (Chraibi and Horisberger 2002; Sheng et al. 2006). A Na^+^ channel tuned to efficiently recover Na^+^ when Na^+^ is scarce, while limiting recovery when Na^+^ is abundant may have provided a selective advantage. Further experimentation will be required to determine whether ENaC Na^+^ self-inhibition has a role in renal physiology.

Na^+^ self-inhibition appears to define ancestral ENaCs. These early channels also had asymmetric ion conduction pathways conferring high Na^+^:K^+^ selectivity, and C-terminal PPxY motifs that facilitate interactions with NEDD4L (Wesch et al. 2010; Hanukoglu and Hanukoglu 2016; Wang, Balchak, et al. 2022). Aldosterone signaling later co-opted NEDD4L-dependent ENaC regulation (Wang, Balchak, et al. 2022). In ENaC expressing fishes, the channel mediates Na^+^ uptake for body fluid homeostasis, and accordingly, its subunits have been found in the gill, kidney, and intestinal tissues (Uchiyama et al. 2012, 2015; Ferreira-Martins et al. 2016; Tseng et al. 2022; Wang, Balchak, et al. 2022). Ray-finned fishes lost ENaC subunits after the divergence of bichirs (Hwang et al. 2011; Wang, Balchak, et al. 2022), possibly because Na^+^ uptake through Na^+^–H^+^ exchanger 3 (NHE3) was more energetically efficient (Tseng et al. 2022). When δ subunits emerged in jawed fishes, the new protein quickly lost its C-terminal PPxY motifs (Wesch et al. 2010; Wang, Balchak, et al. 2022) and later lost Na^+^ self-inhibition. Its tissue distribution also changed. In humans, δ subunit expression is low or absent in aldosterone-sensitive tissues (e.g. kidney and colon), and abundant in the central nervous system (Waldmann et al. 1995; Yamamura et al. 2006; Giraldez et al. 2007; Kashlan et al. 2024). Disparate resting membrane potential requirements in transporting epithelia when compared with excitable cells may alternatively account for the differences in selection we found. ENaC expression increases membrane Na^+^ permeability and promotes depolarization, with Na^+^ self-inhibition limiting the effect at higher Na^+^ concentrations. ENaC-dependent membrane depolarization is important in several contexts, including in the distal nephron, and excitable cells in the lingual epithelium and central nervous system (Gray et al. 2005; Giraldez et al. 2013; Oshima et al. 2013; Nomura et al. 2020). However, in the treeshrew, we detected δ subunit expression in the colon, but not the brain or kidney. Furthermore, the δ subunit was lost altogether in several rodent species (Gettings et al. 2021). In addition, noncanonical ENaC assemblies (e.g. SCNN1D homotrimers) may account for changes in selection; however, little is known about the function or role of such channels (Weisz and Johnson 2003; Ertuglu et al. 2024; Kashlan et al. 2024). Finally, we observed a loss of purifying selection for Na^+^ self-inhibition in the δ subunit but did not find evidence for diversifying selection. We therefore find no evidence that the loss of Na^+^ self-inhibition in the δ subunit increased fitness. Instead, evidence suggests that the loss of Na^+^ self-inhibition in the δ subunit had little impact, in contrast to the α subunit. If the δ subunit has an evolutionarily conserved role in physiology, it remains undefined.

In summary, we conferred a novel function to the ENaC δ subunit, providing evidence that the proposed Na^+^ binding site in the α subunit's periphery is a bona fide allosteric inhibitory effector site. Purifying selection pressure maintained this site in the α subunit but relaxed in the paralogous δ subunit leading to site loss on numerous occasions. This site was likely present in ancient ENaCs when vertebrates first emerged and may be critical to the channel's role in Na^+^ homeostasis.

## Materials and Methods

### Multiple Sequence Alignment and Phylogenetic Tree Calculation

Sequences were retrieved using NCBI's BLAST tool (https://blast.ncbi.nlm.nih.gov/Blast.cgi) and human ENaC α and δ subunit protein sequences (NCBI accession: NP_001029.1, AAI25075.1), resulting in 851 protein sequences (supplementary table S1, Supplementary Material online). Sequences were then aligned using Mafft (Katoh and Standley 2013), and curated using BMGE (Criscuolo and Gribaldo 2010). The phylogenetic tree was calculated from the curated alignment using PhyML (Guindon et al. 2010) and the LG substitution model (Le and Gascuel 2008). The resulting tree (supplementary file S1, Supplementary Material online) was visualized using FigTree (http://tree.bio.ed.ac.uk/software/figtree/) and used in subsequent analyses.

### Testing Phylogenetic Models of Site Gain and Loss

Evolutionary models of trait gain and loss were compared using BayesTraits V3 (https://www.evolution.reading.ac.uk/BayesTraitsV3/BayesTraitsV3.html) (Pagel et al. 2004). Traits were assigned as having or lacking the Asp, as indicated in Fig. 2 (supplementary file S2, Supplementary Material online). Sequences where the key region was missing (gray in Fig. 2) were assigned as unknown. Parameters for each BayesTraits run are provided in supplementary file S3, Supplementary Material online. We compared nested models using a likelihood ratio test, with 1 degree of freedom due to the extra parameter in the dependent model. The likelihood ratio statistic [2 × (log-likelihood⟨dependent model⟩ − log-likelihood⟨independent model⟩)] was converted to a *P*-value using Microsoft Excel and the *chisq.dist.rt* function.

To determine whether the Asp trait was present in an ancestral node, we used BayesTraits to fit the dependent model using MCMC methods while fixing the node of interest to reflect each hypothesis. Parameters for each run are provided in supplementary file S3, Supplementary Material online. Likely average values for model parameters were determined using maximum likelihood runs for each model. As values were within an order of magnitude of 0.1, exponentially distributed priors with a mean of 0.1 were used for all parameters. Runs were performed for the default number of iterations (1,010,000), and resulted in parameter distributions with medians similar to preliminary maximum likelihood runs, consistent with convergence. Log marginal likelihood values were calculated using the stepping stone sampler (100 stones with 10,000 iterations). Evidence for model preference was determined using log Bayes Factors, calculated as: 2 × (log marginal likelihood model 1−log marginal likelihood model 2), where values <2 provide weak evidence, values >2 provide positive evidence, and values >5 provide strong evidence for a model preference.

### dN/dS Measurements

Values for dN and dS were measured and compared using HYPHY's FEL and Contrast-FEL modules implemented at datamonky.org. Codon sequences were retrieved for 458 α and δ subunit sequences, and then aligned and trimmed to the region of interest (supplementary file S4, Supplementary Material online). The phylogenetic relationship of the sequence subset was determined from the protein sequences, as described above, and appended to the sequence file. After uploading sequence and phylogenetic data, the user-defined tree was rerooted on the lamprey α sequence. To compare dN and dS for each subunit, the α subunit or δ subunit branches were selected before running FEL. To compare dN between subunits, α subunit and δ subunit partitions were defined before running Contrast-FEL. Results for FEL and Contrast-FEL runs are provided in supplementary file S4, Supplementary Material online, with summary data provided in Table 1.

### Site-Directed Mutagenesis and ENaC Expression in *Xenopus Oocytes*

Plasmids encoding wild-type human ENaC subunits (hα, hβ, hγ, and hδ) were gifts from Tom Kleyman. A plasmid encoding tδ was synthesized in pTwist CMV plasmid (Twist Bioscience) and moved into psp64 with a C-terminal HA epitope tag. Point mutations were generated using the QuikChange II XL site-directed mutagenesis kit (Agilent, Santa Clara, CA, USA) and confirmed by direct sequencing (GENEWIZ). Wild-type and mutant RNAs were synthesized using mMESSAGE mMACHINE T3, T7 or Sp6 in vitro transcriptional kit (Invitrogen). Oocytes from *X. laevis* frogs were harvested as previously described (Sheng et al. 2005), as approved by the University of Pittsburgh's Institutional Animal Care and Use Committee and were provided by the Pittsburgh Center for Kidney Research. Two nanograms of each ENaC wild-type or mutant subunit were injected into stage V or stage VI oocytes maintained at 18 °C in modified Barth's saline solution: 88 mM NaCl, 1 mM KCl, 2.4 mM NaHCO_3_, 15 mM Hepes, 0.3 mM Ca(NO_3_)_2_, 0.41 mM CaCl_2_, 0.82 mM MgSO_4_, 10 μg/mL streptomycin sulfate, 100 μg/mL gentamycin sulfate, and 10 μg/mL sodium penicillin, pH 7.4.

### Measurement of Na^+^ Self-inhibition by two-electrode Voltage Clamp

Two-electrode voltage-clamp studies were performed at room temperature 24 h after RNA injection using an Axoclamp 900A Computer-Controlled Microelectrode Amplifier and DigiData 1440A interface controlled by pClamp 10.4 (Molecular Devices). Oocytes were placed in a chamber and perfused at constant flow rates (5 mL/min). Glass pipettes filled with 3 M KCl were inserted into oocytes, and the intracellular potential was clamped at −100 mV. Na^+^ self-inhibition measurements were performed as previously described (Kashlan, Kinlough, et al. 2018). Briefly, Na^+^ self-inhibition responses were recorded following a rapid transition from a low [Na^+^] bath solution (1 mM NaCl, 109 mM *N*-methyl-D-glucamine, 2 mM KCl, 2 mM CaCl_2_, and 10 mM HEPES, pH 7.4) to a high [Na^+^] bath solution (110 mM NaCl, 2 mM KCl, 2 mM CaCl_2_, and 10 mm HEPES, pH 7.4). Toward the end of each recording, amiloride (10 µM) was added to define the ENaC-dependent portion of the current. The currents at the peak and at steady state were used to determine the Na^+^ self-inhibition response. Individual data points are shown with summary statistics (mean ± SD). Significance between the two groups was determined by Student's *t*-test and for multiple groups by one-way ANOVA followed by Šidák's multiple comparisons test. *P* < 0.05 was considered significant. All statistical analyses were performed using GraphPad Prism 10.

### Reverse Transcriptase PCR

Northern treeshrew (*T. belangeri*) tissues were harvested from ∼30-week-old animals bred through the University of Alabama at Birmingham (UAB) treeshrew core, as approved by the UAB Institutional Animal Care and Use Committee. *Gallus gallus* tissues were harvested from a female slaughtered at Double R Ranch (New Wilmington, PA, USA). *Marmosa mexicana* kidney tissue was acquired through the Carnegie Museum of Natural History, which was collected from an adult female specimen in Izabel, Guatemala, at 15°40′N, 88°41′W on 1994 June 13 and stored at −80 °C.

Total RNA was extracted from ∼50 mg tissue using TRIzol reagent (Invitrogen). cDNA was generated using the RevertAid Reverse Transcriptase Kit (ThermoFisher Scientific). Specific primers for ENaC subunits and glyceraldehyde-3-phosphate dehydrogenase (GAPDH) as an internal control (supplementary table S3, Supplementary Material online) were designed using NCBI primer-blast tools. PCR was run for 30 cycles using GoTaq G2 Green master mix (Promega) and specific primers, with the annealing temperature set at 58 °C. PCR products were visualized with GelRed dye (Biotium) after agarose gel electrophoresis using a GelDoc imaging system (BioRad).

### Analysis of Gene Expression Omnibus Datasets

Expression of ENaC subunits in *A. carolinensis* tissues was assessed from Gene Expression Omnibus dataset GSE97367, Convergent origination of a Drosophila-like dosage compensation mechanism in a reptile lineage (gene expression profiling in several tetrapod species, bulk tissue RNA-seq) (Marin et al. 2017). The *Anolis* FPKM data file was downloaded and the reported *scnn1a*, *scnn1d*, *scnn1b*, and *scnn1g* expression data was retrieved by using HCOP:Orthology Predictions Search tool from www.genenames.org to convert the gene symbols into Ensembl gene IDs. Genes with FPKM >0.3 were considered to be “expressed” (Hart et al. 2013).

Expression of ENaC subunits in *A. cygnoides* renal tissue were assessed from Gene Expression Omnibus dataset GSE144723, RNA-seq Analysis on the Renal Tissue Injury in the Development of Goslings Gout (study unpublished). Fastq files for the six healthy samples were downloaded and aligned to the *Anser cynoides* reference genome, GooseV1.0 (RefSeq GCF_002166845.1) using STAR aligner (Dobin et al. 2013). Gene expression feature counts were generated using the *featureCounts* function for the Rsubread package in R (Liao et al. 2019; R Core Team 2021) and RPKM was calculated using the *rpkm* function from edgeR (Robinson et al. 2010). Genes with RPKM>0.3 were considered to be “expressed” (Ramskold et al. 2009).

## Supplementary Material

msae162_Supplementary_Data

## Data Availability

Data for electrophysiology and uncropped gels are provided as source data. Primer sequences are provided as a supplementary file, Supplementary Material online. Sequencing data for *Marmosa mexicana* kidney tissue are available at NCBI (BioProject PRJNA1052681). Input files and parameters used for phylogenetic trait analysis are provided as supplementary files, Supplementary Material online.
