# Supplementary material for: Varying Selection Pressure for a Na+ Sensing Site in Epithelial Na+ Channel Subunits Reflect Divergent Roles in Na+ Homeostasis
Source: Mol Biol Evol. 2024 Aug 5;41(8):msae162. doi: 10.1093/molbev/msae162 (PMC11331422; doi:10.1093/molbev/msae162)

treeshrew α (373 bp) and β (206 bp)

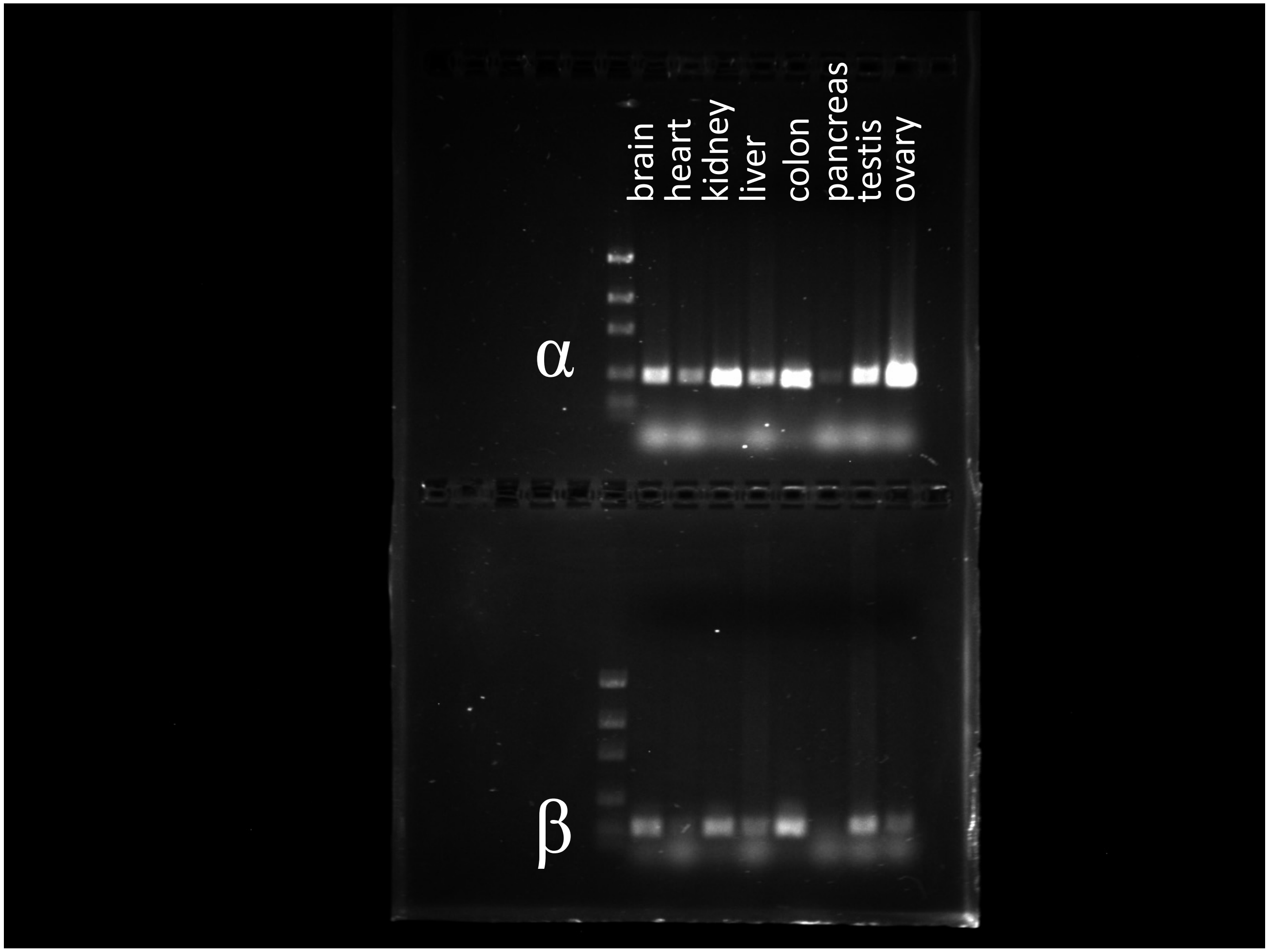

treeshrew γ (260 bp)

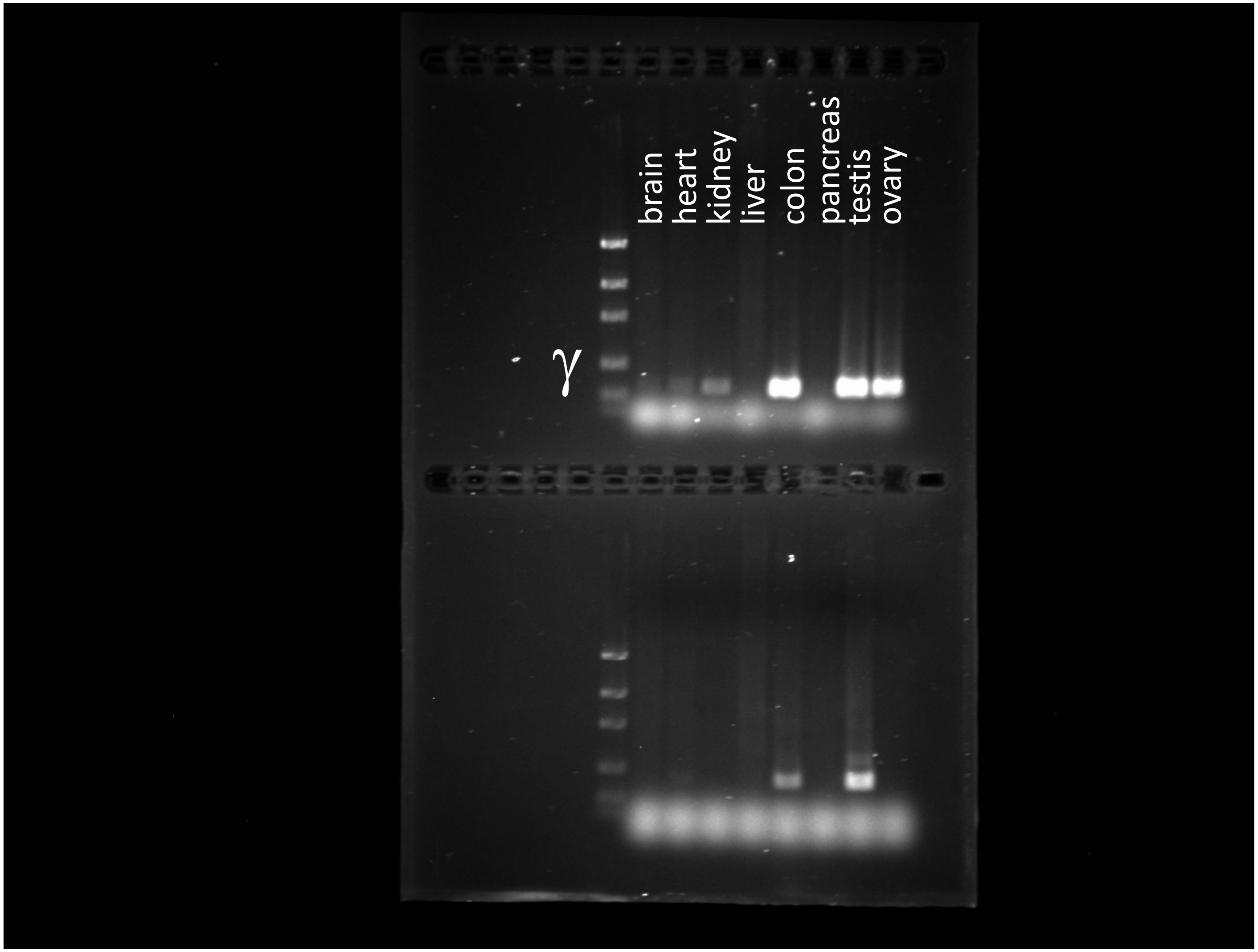

treeshrew δ (802 bp)

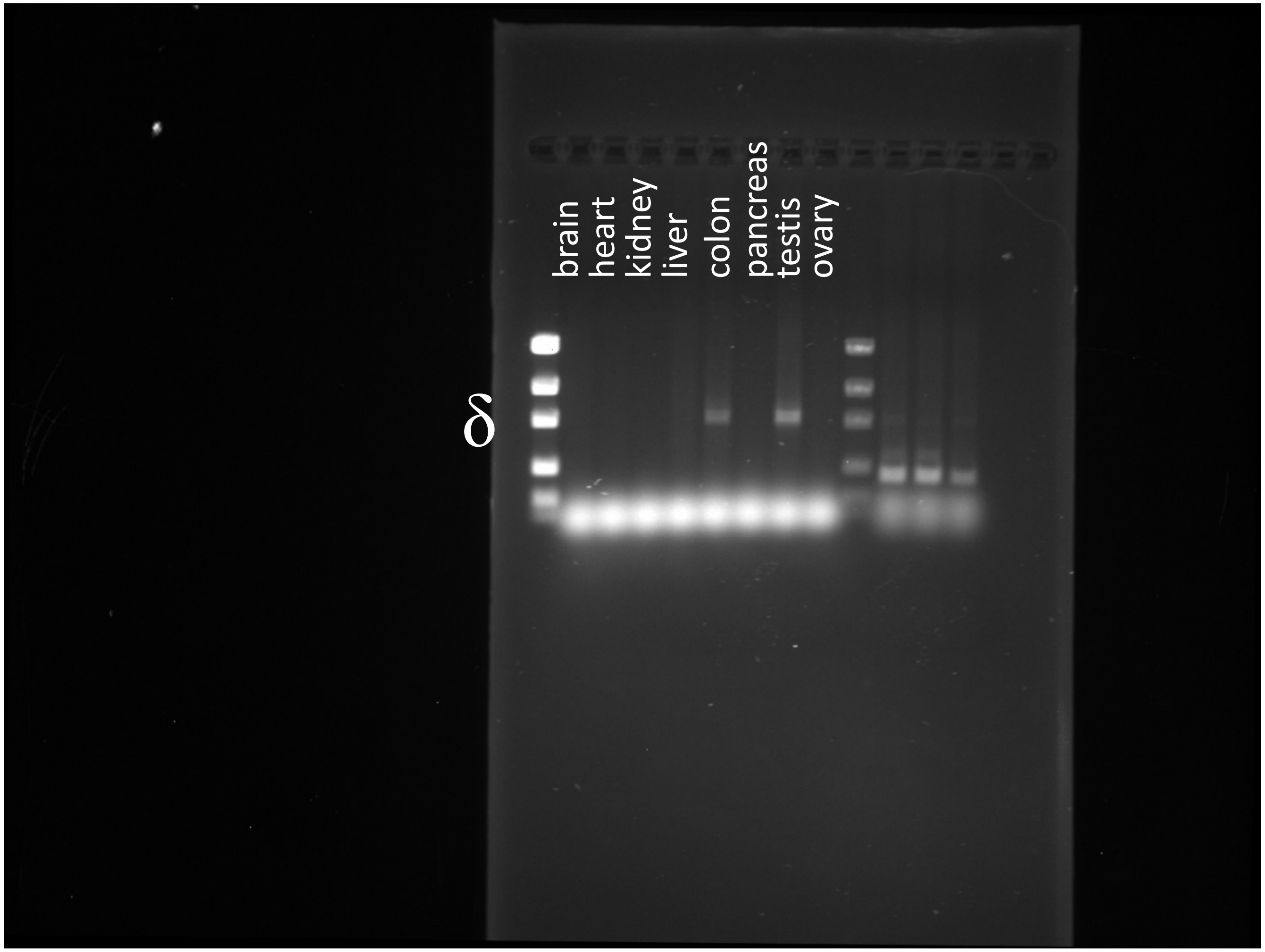

treeshrew GAPDH (378 bp)

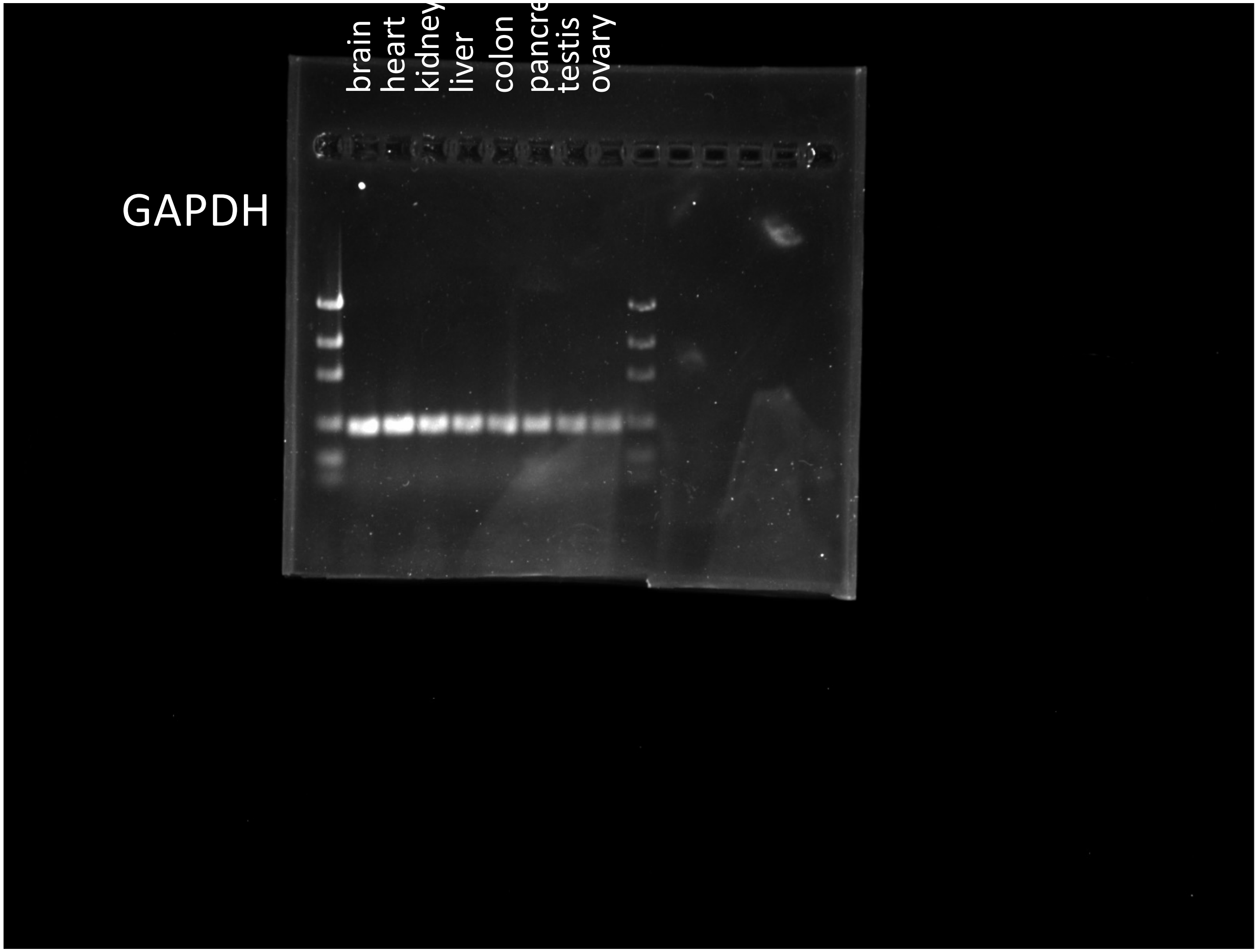

treeshrew negative controls

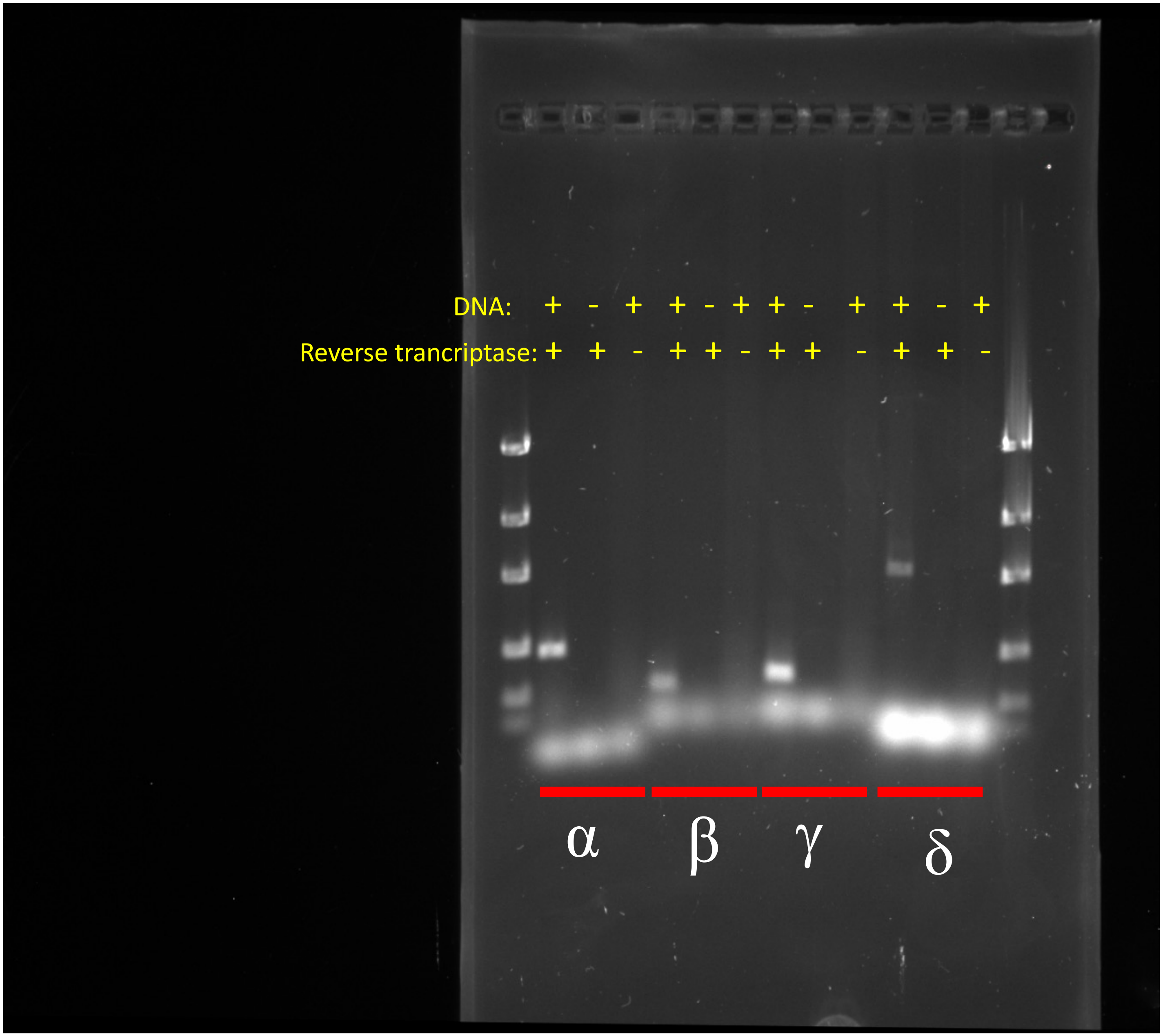

α, β and γ: kidney

δ: testis

chicken α (939 bp)

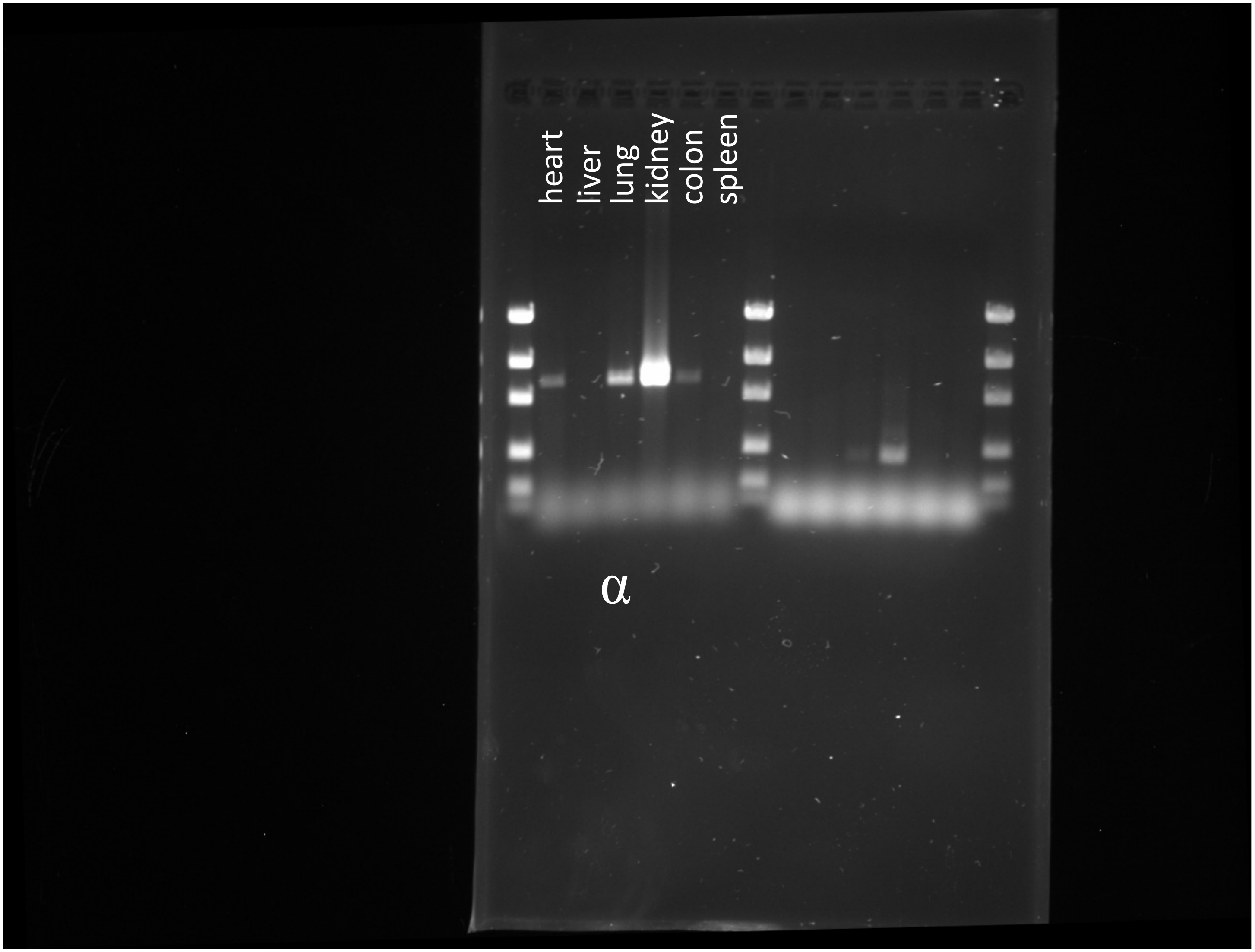

chicken β (340 bp)

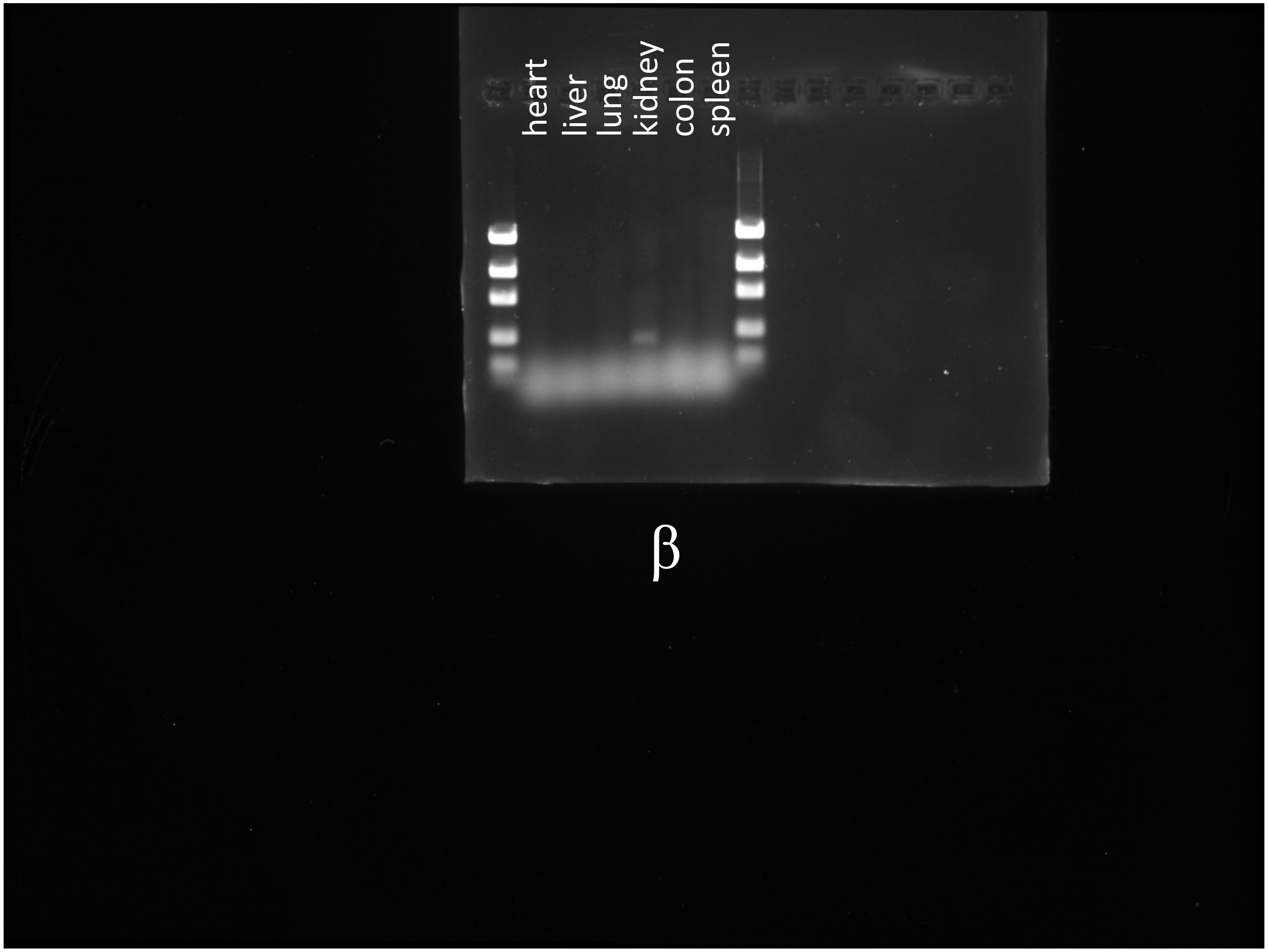

chicken γ (621 bp) and δ (387 bp)

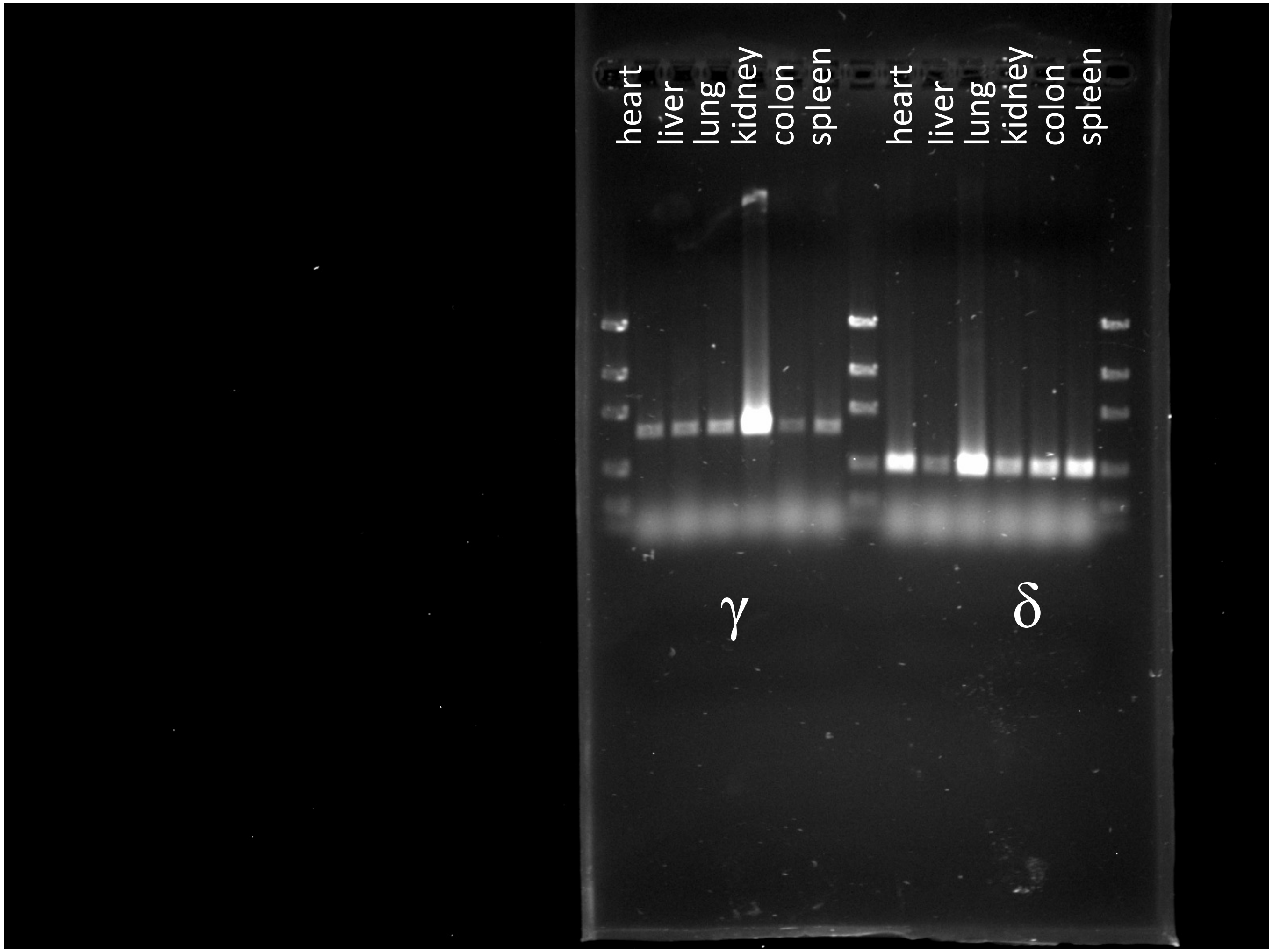

chicken GAPDH (540 bp)

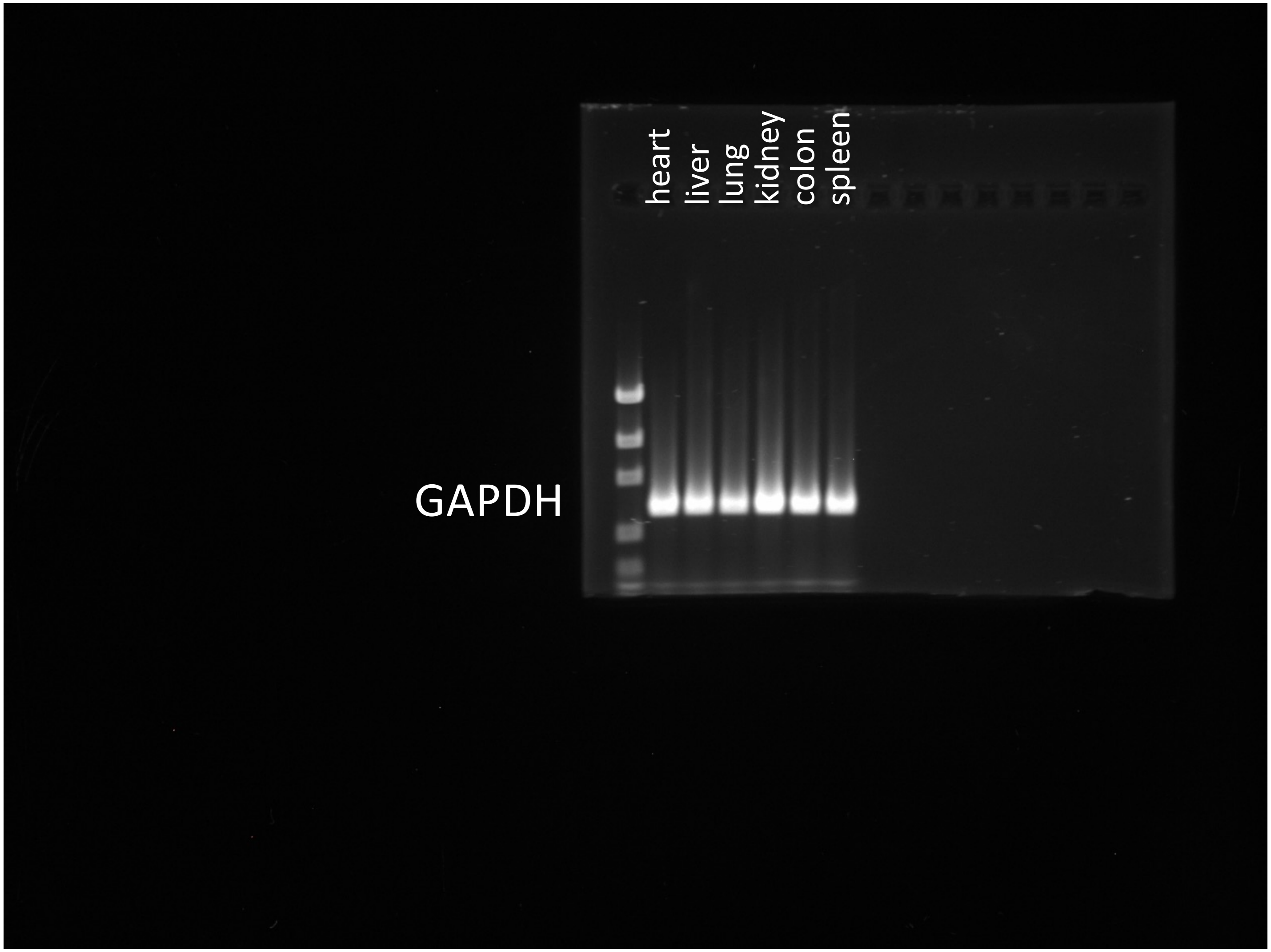

chicken negative controls

α, β, γ and δ : kidney

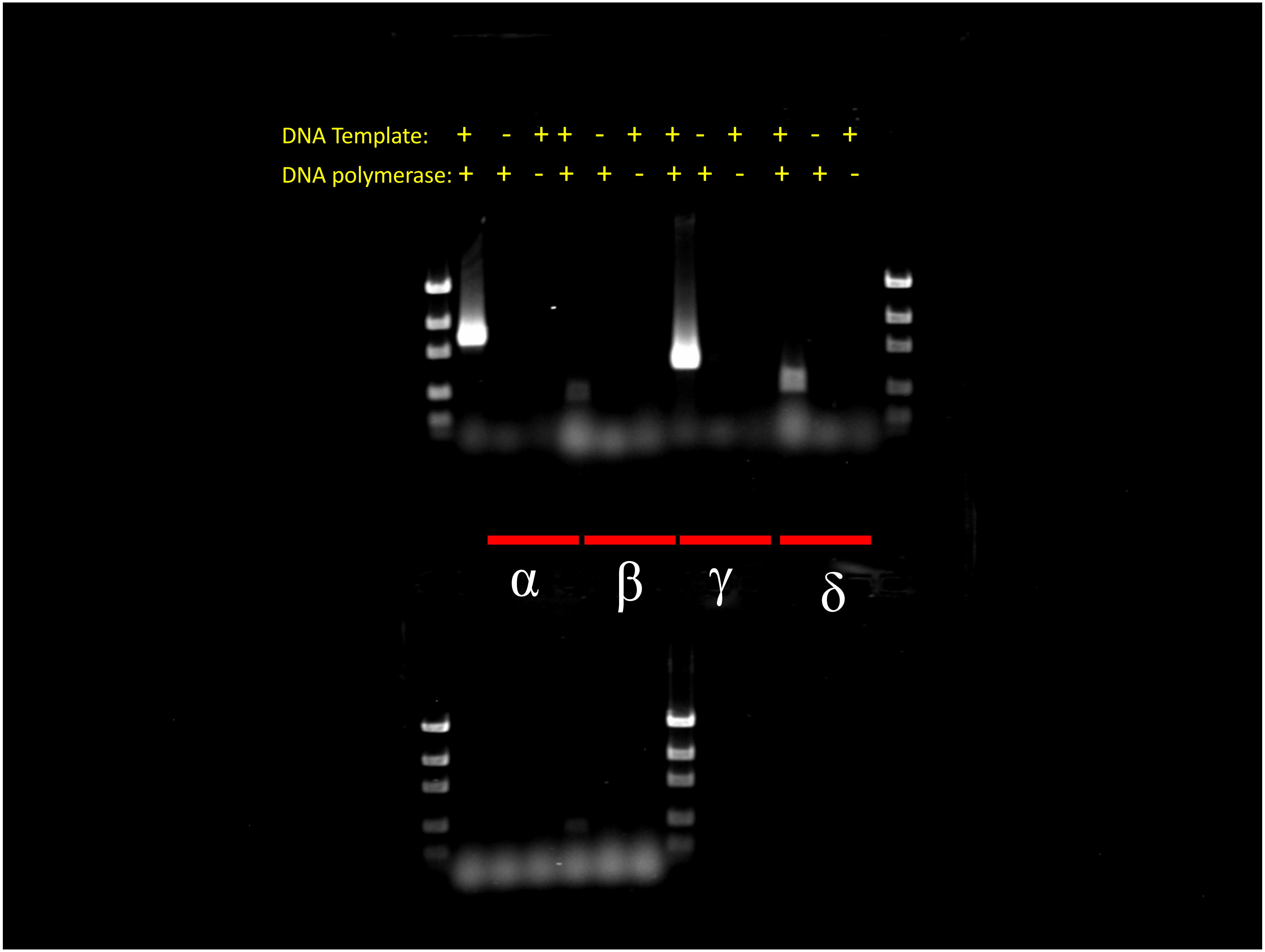

Supplement: msae162_Supplementary_Data [file msae162_supplementary_data.zip › Figure 5-source data.pdf]
