# Supplementary material for: Varying Selection Pressure for a Na+ Sensing Site in Epithelial Na+ Channel Subunits Reflect Divergent Roles in Na+ Homeostasis
Source: Mol Biol Evol. 2024 Aug 5;41(8):msae162. doi: 10.1093/molbev/msae162 (PMC11331422; doi:10.1093/molbev/msae162)
Supplement: msae162_Supplementary_Data [file msae162_supplementary_data.zip › Supplementary Table 1.pdf]

Class	Order	Family	Species Name	ENaC subunit	Accession
Mammalia	Primates	Hominidae	*Homo Sapiens*	SCNN1B	NP_000327.2
Mammalia	Primates	Hominidae	*Homo Sapiens*	SCNN1G	NP_001030.2
Hyperoartia	Petromyzontiformes	Petromyzontidae	*Petromyzon marinus*	SCNN1A	XP_032824081.1
Myxini	Myxiniformes	Myxinidae	*Eptatretus burgeri*	SCNN1A	ENSEBUT00000015784.1
Chondrichthyes	Orectolobiformes	Hemiscylliidae	*Chiloscyllium plagiosum*	SCNN1A	XP_043538348.1
Chondrichthyes	Carcharhiniformes	Scyliorhinidae	*Scyliorhinus canicula*	SCNN1A	XP_038629974.1
Chondrichthyes	Lamniformes	Lamnidae	*Carcharodon carcharias*	SCNN1A	XP_041036036.1
Actinopteri	Polypteriformes	Polypteridae	*Erpetoichthys calabaricus*	SCNN1A	XP_028666007.1
Actinopteri	Polypteriformes	Polypteridae	*Polypterus senegalus*	SCNN1A	XP_039619758.1
Sarcopterygii	Coelacanthiformes	Latimeriidae	*Latimeria chalumnae*	SCNN1A	XP_006009076.1
Amphibia	Gymnophiona	Dermophiidae	*Geotrypetes seraphini*	SCNN1A	XP_033778898.1
Amphibia	Gymnophiona	Rhinatrematidae	*Rhinatrema bivittatum*	SCNN1A	XP_029436262.1
Amphibia	Gymnophiona	Siphonopidae	*Microcaecilia unicolor*	SCNN1A	XP_030043259.1
Amphibia	Anura	Bufonidae	*Rhinella marina*	SCNN1A	BAE16707.1
Amphibia	Anura	Dendrobatidae	*Ranitomeya imitator*	SCNN1A	CAF4873988.1
Amphibia	Anura	Dicroglossidae	*Nanorana parkeri*	SCNN1A	XP_018422874.1
Amphibia	Anura	Pipidae	*Xenopus laevis*	SCNN1A	AAI69904.1
Amphibia	Anura	Pipidae	*Xenopus tropicalis*	SCNN1A	NP_001184124.1
Amphibia	Anura	Ranidae	*Lithobates catesbeianus*	SCNN1A	AAM53957.1
Reptilia	Squamata	Agamidae	*Pogona vitticeps*	SCNN1A	XP_020659815.1
Reptilia	Squamata	Colubridae	*Thamnophis elegans*	SCNN1A	XP_032068406.1
Reptilia	Squamata	Colubridae	*Pantherophis guttatus*	SCNN1A	XP_034266825.1
Reptilia	Squamata	Dactyloidae	*Anolis carolinensis*	SCNN1A	XP_003226512.1
Reptilia	Squamata	Elapidae	*Ophiophagus hannah*	SCNN1A	ETE61106.1
Reptilia	Squamata	Elapidae	*Notechis scutatus*	SCNN1A	XP_026543814.1
Reptilia	Squamata	Elapidae	*Pseudonaja textilis*	SCNN1A	XP_026569126.1
Reptilia	Squamata	Gekkonidae	*Paroedura picta*	SCNN1A	GCF53442.1
Reptilia	Squamata	Lacertidae	*Podarcis muralis*	SCNN1A	XP_028568612.1
Reptilia	Squamata	Lacertidae	*Zootoca vivipara*	SCNN1A	XP_034953096.1
Reptilia	Squamata	Pythonidae	*Python bivittatus*	SCNN1A	XP_007429678.2
Reptilia	Squamata	Viperidae	*Protobothrops mucrosquamatus*	SCNN1A	XP_015669540.1
Reptilia	Squamata	Viperidae	*Crotalus tigris*	SCNN1A	XP_039194635.1
Reptilia	Testudines	Cheloniidae	*Chelonia mydas*	SCNN1A	XP_007061445.2
Reptilia	Testudines	Dermochelyidae	*Dermochelys coriacea*	SCNN1A	XP_038237693.1
Reptilia	Testudines	Emydidae	*Terrapene carolina triunguis*	SCNN1A	XP_024067843.1
Reptilia	Testudines	Emydidae	*Trachemys scripta elegans*	SCNN1A	XP_034643586.1
Reptilia	Testudines	Geoemydidae	*Mauremys reevesii*	SCNN1A	XP_039376257.1
Reptilia	Testudines	Kinosternidae	*Platysternon megacephalum*	SCNN1A	TFK01472.1
Reptilia	Testudines	Testudinidae	*Gopherus evgoodei*	SCNN1A	XP_030391202.1

Class	Order	Family	Species Name	ENaC subunit	Accession
Reptilia	Testudines	Testudinidae	*Chelonoidis abingdonii*	SCNN1A	XP_032621253.1
Reptilia	Testudines	Trionychidae	*Pelodiscus sinensis*	SCNN1A	XP_006124236.1
Reptilia	Crocodilia	Alligatoridae	*Alligator mississippiensis*	SCNN1A	KYO22283.1
Reptilia	Crocodilia	Alligatoridae	*Alligator sinensis*	SCNN1A	XP_025067723.1
Reptilia	Crocodilia	Crocodylidae	*Crocodylus porosus*	SCNN1A	XP_019404508.1
Reptilia	Crocodilia	Gavialidae	*Gavialis gangeticus*	SCNN1A	XP_019382935.1
Aves	Struthioniformes	Struthionidae	*Struthio camelus australis*	SCNN1A	KFV86516.1
Aves	Tinamiformes	Tinamidae	*Tinamus guttatus*	SCNN1A	KGL82287.1
Aves	Tinamiformes	Tinamidae	*Crypturellus soui*	SCNN1A	NWI14039.1
Aves	Tinamiformes	Tinamidae	*Crypturellus undulatus*	SCNN1A	NWJ02209.1
Aves	Tinamiformes	Tinamidae	*Nothoprocta ornata*	SCNN1A	NWY02795.1
Aves	Tinamiformes	Tinamidae	*Nothocercus julius*	SCNN1A	NXA53269.1
Aves	Tinamiformes	Tinamidae	*Nothocercus nigrocapillus*	SCNN1A	NXD14816.1
Aves	Tinamiformes	Tinamidae	*Nothoprocta perdicaria*	SCNN1A	XP_025910087.1
Aves	Apterygiformes	Apterygidae	*Apteryx mantelli mantelli*	SCNN1A	XP_013813503.1
Aves	Casuariiformes	Casuariidae	*Casuarius casuarius*	SCNN1A	NXE54182.1
Aves	Casuariiformes	Dromaiidae	*Dromaius novaehollandiae*	SCNN1A	NXG34143.1
Aves	Anseriformes	Anatidae	*Nipponia nippon*	SCNN1A	KFQ90921.1
Aves	Anseriformes	Anatidae	*Anas platyrhynchos*	SCNN1A	XP_027305532.1
Aves	Anseriformes	Anatidae	*Aythya fuligula*	SCNN1A	XP_032063342.1
Aves	Anseriformes	Anatidae	*Oxyura jamaicensis*	SCNN1A	XP_035170163.1
Aves	Anseriformes	Anatidae	*Cygnus atratus*	SCNN1A	XP_035427469.1
Aves	Anseriformes	Anatidae	*Cygnus olor*	SCNN1A	XP_040429005.1
Aves	Anseriformes	Anseranatidae	*Anseranas semipalmata*	SCNN1A	NXI67393.1
Aves	Galliformes	Megapodiidae	*Alectura lathami*	SCNN1A	NXL83370.1
Aves	Galliformes	Numididae	*Numida meleagris*	SCNN1A	XP_021252254.1
Aves	Galliformes	Odontophoridae	*Odontophorus gujanensis*	SCNN1A	NXJ09915.1
Aves	Galliformes	Odontophoridae	*Colinus virginianus*	SCNN1A	OXB76261.1
Aves	Galliformes	Phasianidae	*Meleagris gallopavo*	SCNN1A	XP_010716384.1
Aves	Galliformes	Phasianidae	*Phasianus colchicus*	SCNN1A	XP_031466087.1
Aves	Galliformes	Phasianidae	*Gallus gallus*	SCNN1A	XP_040514545.1
Aves	Podicipediformes	Podicipedidae	*Podiceps cristatus*	SCNN1A	KFZ55580.1
Aves	Podicipediformes	Podicipedidae	*Podilymbus podiceps*	SCNN1A	NXL46488.1
Aves	Columbiformes	Columbidae	*Columbina picui*	SCNN1A	NWQ80800.1
Aves	Columbiformes	Columbidae	*Caloenas nicobarica*	SCNN1A	NWX04980.1
Aves	Columbiformes	Columbidae	*Alopecoenas beccarii*	SCNN1A	NXW92259.1
Aves	Columbiformes	Columbidae	*Patagioenas fasciata monilis*	SCNN1A	OPJ69346.1
Aves	Columbiformes	Columbidae	*Columba livia*	SCNN1A	XP_005506326.1
Aves	Pterocliformes	Pteroclididae	*Pterocles burchelli*	SCNN1A	NWU63913.1

Class	Order	Family	Species Name	ENaC subunit	Accession
Aves	Pterocliformes	Pteroclididae	*Pterocles gutturalis*	SCNN1A	XP_010079085.1
Aves	Mesitornithiformes	Mesitornithidae	*Mesitornis unicolor*	SCNN1A	XP_010180376.1
Aves	Cuculiformes	Cuculidae	*Cuculus canorus*	SCNN1A	KFO80481.1
Aves	Cuculiformes	Cuculidae	*Geococcyx californianus*	SCNN1A	NWH66415.1
Aves	Cuculiformes	Cuculidae	*Piaya cayana*	SCNN1A	NWH73335.1
Aves	Cuculiformes	Cuculidae	*Centropus unirufus*	SCNN1A	NWR77714.1
Aves	Cuculiformes	Cuculidae	*Crotophaga sulcirostris*	SCNN1A	NWS70893.1
Aves	Cuculiformes	Cuculidae	*Centropus bengalensis*	SCNN1A	NXX98496.1
Aves	Musophagiformes	Musophagidae	*Tauraco erythrolophus*	SCNN1A	KFV03165.1
Aves	Musophagiformes	Musophagidae	*Corythaeola cristata*	SCNN1A	NXC21801.1
Aves	Musophagiformes	Musophagidae	*Corythaixoides concolor*	SCNN1A	NXJ94082.1
Aves	Caprimulgiformes	Aegothelidae	*Aegotheles bennettii*	SCNN1A	NWX11496.1
Aves	Caprimulgiformes	Caprimulgidae	*Antrostomus carolinensis*	SCNN1A	KFZ63672.1
Aves	Caprimulgiformes	Nyctibiidae	*Nyctibius bracteatus*	SCNN1A	NXF36768.1
Aves	Caprimulgiformes	Steatornithidae	*Steatornis caripensis*	SCNN1A	NWX48982.1
Aves	Opisthocomiformes	Opisthocomidae	*Opisthocomus hoazin*	SCNN1A	XP_009930503.1
Aves	Gruiformes	Aramidae	*Aramus guarauna*	SCNN1A	NXO55305.1
Aves	Gruiformes	Gruidae	*Balearica regulorum gibbericeps*	SCNN1A	KFO05370.1
Aves	Gruiformes	Gruidae	*Grus americana*	SCNN1A	NWH22444.1
Aves	Gruiformes	Heliornithidae	*Heliornis fulica*	SCNN1A	NXP56696.1
Aves	Gruiformes	Otididae	*Ardeotis kori*	SCNN1A	NXE26039.1
Aves	Gruiformes	Psophiidae	*Psophia crepitans*	SCNN1A	NXI91101.1
Aves	Gruiformes	Rallidae	*Zapornia atra*	SCNN1A	NXT82576.1
Aves	Gruiformes	Rallidae	*Ceuthmochares aereus*	SCNN1A	NXY47806.1
Aves	Gruiformes	Rhynochetidae	*Rhynochetos jubatus*	SCNN1A	NWW84520.1
Aves	Charadriiformes	Alcidae	*Alca torda*	SCNN1A	NWX74339.1
Aves	Charadriiformes	Alcidae	*Cepphus grylle*	SCNN1A	NXV19368.1
Aves	Charadriiformes	Alcidae	*Uria aalge*	SCNN1A	NXV47839.1
Aves	Charadriiformes	Burhinidae	*Burhinus bistriatus*	SCNN1A	NWQ88663.1
Aves	Charadriiformes	Charadriidae	*Charadrius vociferus*	SCNN1A	KGL87116.1
Aves	Charadriiformes	Chionidae	*Chionis minor*	SCNN1A	NWY49179.1
Aves	Charadriiformes	Chionidae	*Eudromia elegans*	SCNN1A	NXA41911.1
Aves	Charadriiformes	Dromadidae	*Dromas ardeola*	SCNN1A	NWU46356.1
Aves	Charadriiformes	Glareolidae	*Rhinoptilus africanus*	SCNN1A	NXN46301.1
Aves	Charadriiformes	Glareolidae	*Glareola pratincola*	SCNN1A	NXY79587.1
Aves	Charadriiformes	Jacanidae	*Jacana jacana*	SCNN1A	NXT01513.1
Aves	Charadriiformes	Laridae	*Rynchops niger*	SCNN1A	NXN53830.1
Aves	Charadriiformes	Laridae	*Rissa tridactyla*	SCNN1A	NXV36933.1
Aves	Charadriiformes	Laridae	*Phaetusa simplex*	SCNN1A	NXW31000.1

Class	Order	Family	Species Name	ENaC subunit	Accession
Aves	Charadriiformes	Laridae	*Larus smithsonianus*	SCNN1A	NXX00132.1
Aves	Charadriiformes	Otididae	*Chlamydotis macqueenii*	SCNN1A	KFP44851.1
Aves	Charadriiformes	Pedionomidae	*Pedionomus torquatus*	SCNN1A	NWW53103.1
Aves	Charadriiformes	Pluvianellidae	*Pluvianellus socialis*	SCNN1A	NXT47411.1
Aves	Charadriiformes	Pteroclididae	*Syrrhaptes paradoxus*	SCNN1A	NXT19455.1
Aves	Charadriiformes	Recurvirostridae	*Himantopus himantopus*	SCNN1A	NXN72093.1
Aves	Charadriiformes	Scolopacidae	*Arenaria interpres*	SCNN1A	NXK19429.1
Aves	Charadriiformes	Scolopacidae	*Limosa lapponica baueri*	SCNN1A	PKU37096.1
Aves	Charadriiformes	Stercorariidae	*Stercorarius parasiticus*	SCNN1A	NXG83226.1
Aves	Charadriiformes	Thinocoridae	*Thinocorus orbignyianus*	SCNN1A	NXP05103.1
Aves	Charadriiformes	Turnicidae	*Turnix velox*	SCNN1A	NXU50974.1
Aves	Apodiformes	Apodidae	*Chaetura pelagica*	SCNN1A	KFU84168.1
Aves	Apodiformes	Apodidae	*Nyctiprogne leucopyga*	SCNN1A	NXW41374.1
Aves	Apodiformes	Hemiprocnidae	*Hemiprocne comata*	SCNN1A	NXG55329.1
Aves	Apodiformes	Trochilidae	*Oreotrochilus melanogaster*	SCNN1A	NXU73667.1
Aves	Apodiformes	Trochilidae	*Calypte anna*	SCNN1A	XP_008503681.2
Aves	Eurypygiformes	Eurypygidae	*Eurypyga helias*	SCNN1A	KFW00191.1
Aves	Phaethontiformes	Phaethontidae	*Phaethon lepturus*	SCNN1A	XP_010295848.1
Aves	Gaviiformes	Gaviidae	*Gavia stellata*	SCNN1A	KFV55930.1
Aves	Sphenisciformes	Spheniscidae	*Megadyptes antipodes antipodes*	SCNN1A	KAF1469511.1
Aves	Sphenisciformes	Spheniscidae	*Eudyptula albosignata*	SCNN1A	KAF1511665.1
Aves	Sphenisciformes	Spheniscidae	*Eudyptes sclateri*	SCNN1A	KAF1519152.1
Aves	Sphenisciformes	Spheniscidae	*Eudyptes pachyrhynchus*	SCNN1A	KAF1598945.1
Aves	Sphenisciformes	Spheniscidae	*Pygoscelis papua*	SCNN1A	KAF1673280.1
Aves	Sphenisciformes	Spheniscidae	*Pygoscelis adeliae*	SCNN1A	XP_009327297.1
Aves	Ciconiiformes	Ciconiidae	*Ciconia maguari*	SCNN1A	NXJ43793.1
Aves	Suliformes	Anhingidae	*Anhinga rufa*	SCNN1A	NXT87286.1
Aves	Suliformes	Fregatidae	*Fregata magnificens*	SCNN1A	NWH45437.1
Aves	Suliformes	Phalacrocoracidae	*Phalacrocorax carbo*	SCNN1A	XP_009510002.1
Aves	Suliformes	Sulidae	*Sula dactylatra*	SCNN1A	NWI24873.1
Aves	Procellariiformes	Hydrobatidae	*Oceanites oceanicus*	SCNN1A	NXF45845.1
Aves	Procellariiformes	Hydrobatidae	*Oceanodroma tethys*	SCNN1A	NXH73310.1
Aves	Procellariiformes	Procellariidae	*Calonectris borealis*	SCNN1A	NXV91171.1
Aves	Procellariiformes	Procellariidae	*Fulmarus glacialis*	SCNN1A	XP_009585900.1
Aves	Pelecaniformes	Ardeidae	*Egretta garzetta*	SCNN1A	KFP16250.1
Aves	Pelecaniformes	Ardeidae	*Cochlearius cochlearius*	SCNN1A	NXE81667.1
Aves	Pelecaniformes	Balaenicipitidae	*Balaeniceps rex*	SCNN1A	NXS50186.1
Aves	Pelecaniformes	Scopidae	*Scopus umbretta*	SCNN1A	NXX53574.1
Aves	Pelecaniformes	Threskiornithidae	*Ibidorhyncha struthersii*	SCNN1A	NXA23828.1

Class	Order	Family	Species Name	ENaC subunit	Accession
Aves	Strigiformes	Strigidae	*Ciccaba nigrolineata*	SCNN1A	NXF70318.1
Aves	Strigiformes	Strigidae	*Glaucidium brasilianum*	SCNN1A	NXL30616.1
Aves	Strigiformes	Strigidae	*Athene cunicularia*	SCNN1A	XP_026716676.1
Aves	Strigiformes	Tytonidae	*Tyto alba alba*	SCNN1A	XP_009967902.2
Aves	Accipitriformes	Accipitridae	*Haliaeetus albicilla*	SCNN1A	NWZ55270.1
Aves	Accipitriformes	Accipitridae	*Spizaetus tyrannus*	SCNN1A	NXJ44711.1
Aves	Accipitriformes	Accipitridae	*Aquila chrysaetos chrysaetos*	SCNN1A	XP_029876229.1
Aves	Accipitriformes	Pandionidae	*Pandion haliaetus*	SCNN1A	NXS71236.1
Aves	Coliiformes	Coliidae	*Colius striatus*	SCNN1A	KFP26484.1
Aves	Coliiformes	Coliidae	*Urocolius indicus*	SCNN1A	NXX75583.1
Aves	Trogoniformes	Trogonidae	*Trogon melanurus*	SCNN1A	NXJ78550.1
Aves	Bucerotiformes	Bucerotidae	*Buceros rhinoceros silvestris*	SCNN1A	XP_010133519.1
Aves	Bucerotiformes	Bucorvidae	*Bucorvus abyssinicus*	SCNN1A	NWR65272.1
Aves	Bucerotiformes	Upupidae	*Upupa epops*	SCNN1A	NWU98705.1
Aves	Piciformes	Bucconidae	*Xiphorhynchus elegans*	SCNN1A	NXU83966.1
Aves	Piciformes	Galbulidae	*Galbula dea*	SCNN1A	NXI35165.1
Aves	Piciformes	Indicatoridae	*Indicator maculatus*	SCNN1A	NXN10718.1
Aves	Piciformes	Lybiidae	*Tricholaema leucomelas*	SCNN1A	NXX46869.1
Aves	Piciformes	Megalaimidae	*Psilopogon haemacephalus*	SCNN1A	NXG48267.1
Aves	Piciformes	Picidae	*Dryobates pubescens*	SCNN1A	XP_009897900.1
Aves	Piciformes	Ramphastidae	*Eubucco bourcierii*	SCNN1A	NXF94041.1
Aves	Coraciiformes	Alcedinidae	*Halcyon senegalensis*	SCNN1A	NXD85144.1
Aves	Coraciiformes	Alcedinidae	*Chloroceryle aenea*	SCNN1A	NXI56916.1
Aves	Coraciiformes	Alcedinidae	*Eurystomus gularis*	SCNN1A	NXW59852.1
Aves	Coraciiformes	Leptosomatidae	*Leptosomus discolor*	SCNN1A	KFQ09921.1
Aves	Coraciiformes	Meropidae	*Merops nubicus*	SCNN1A	KFQ18174.1
Aves	Coraciiformes	Meropidae	*Rhinopomastus cyanomelas*	SCNN1A	NXN91853.1
Aves	Cariamiformes	Cariamidae	*Cariama cristata*	SCNN1A	XP_009693526.1
Aves	Falconiformes	Falconidae	*Falco peregrinus*	SCNN1A	XP_027639571.1
Aves	Falconiformes	Sagittariidae	*Sagittarius serpentarius*	SCNN1A	NXR00828.1
Aves	Psittaciformes	Cacatuidae	*Probosciger aterrimus*	SCNN1A	NWS46959.1
Aves	Psittaciformes	Psittacidae	*Amazona aestiva*	SCNN1A	KQK78168.1
Aves	Psittaciformes	Psittacidae	*Chunga burmeisteri*	SCNN1A	NWS51256.1
Aves	Psittaciformes	Psittacidae	*Eolophus roseicapilla*	SCNN1A	NXD62470.1
Aves	Psittaciformes	Psittacidae	*Amazona guildingii*	SCNN1A	NXK71394.1
Aves	Psittaciformes	Psittaculidae	*Nestor notabilis*	SCNN1A	KFQ47349.1
Aves	Psittaciformes	Psittaculidae	*Melopsittacus undulatus*	SCNN1A	XP_005146707.3
Aves	Psittaciformes	Strigopidae	*Strigops habroptila*	SCNN1A	XP_030327486.1
Aves	Passeriformes	Acanthizidae	*Daphoenositta chrysoptera*	SCNN1A	NWV55368.1

Class	Order	Family	Species Name	ENaC subunit	Accession
Aves	Passeriformes	Acrocephalidae	*Acrocephalus arundinaceus*	SCNN1A	NWZ71749.1
Aves	Passeriformes	Acrocephalidae	*Hippolais icterina*	SCNN1A	NXR47386.1
Aves	Passeriformes	Aegithalidae	*Aegithalos caudatus*	SCNN1A	NWH88031.1
Aves	Passeriformes	Alcippeidae	*Serilophus lunatus*	SCNN1A	NXM73179.1
Aves	Passeriformes	Bombycillidae	*Urocynchramus pylzowi*	SCNN1A	NWT97823.1
Aves	Passeriformes	Bombycillidae	*Bombycilla garrulus*	SCNN1A	NXN88056.1
Aves	Passeriformes	Buphagidae	*Buphagus erythrorhynchus*	SCNN1A	NXU03492.1
Aves	Passeriformes	Callaeatidae	*Callaeas wilsoni*	SCNN1A	NXY66931.1
Aves	Passeriformes	Calyptophilidae	*Smithornis capensis*	SCNN1A	NXF08430.1
Aves	Passeriformes	Cardinalidae	*Cardinalis cardinalis*	SCNN1A	NWT27697.1
Aves	Passeriformes	Cardinalidae	*Passerina amoena*	SCNN1A	NXP92782.1
Aves	Passeriformes	Certhiidae	*Certhia brachydactyla*	SCNN1A	NXO91443.1
Aves	Passeriformes	Cettiidae	*Hylia prasina*	SCNN1A	NWU43289.1
Aves	Passeriformes	Cettiidae	*Erpornis zantholeuca*	SCNN1A	NXS84048.1
Aves	Passeriformes	Cettiidae	*Cettia cetti*	SCNN1A	NXU98617.1
Aves	Passeriformes	Chloropseidae	*Chloropsis cyanopogon*	SCNN1A	NXP56976.1
Aves	Passeriformes	Cinclidae	*Cinclus mexicanus*	SCNN1A	NXR18441.1
Aves	Passeriformes	Cisticolidae	*Erythrocercus mccallii*	SCNN1A	NWT60588.1
Aves	Passeriformes	Cisticolidae	*Aleadryas rufinucha*	SCNN1A	NXC54822.1
Aves	Passeriformes	Cisticolidae	*Cisticola juncidis*	SCNN1A	NXO24742.1
Aves	Passeriformes	Climacteridae	*Climacteris rufus*	SCNN1A	NWW69788.1
Aves	Passeriformes	Corvidae	*Corvus brachyrhynchos*	SCNN1A	KFO57758.1
Aves	Passeriformes	Corvidae	*Aphelocoma coerulescens*	SCNN1A	NWY21200.1
Aves	Passeriformes	Corvidae	*Corvus moneduloides*	SCNN1A	NXD54317.1
Aves	Passeriformes	Corvidae	*Corvus cornix cornix*	SCNN1A	XP_010394388.2
Aves	Passeriformes	Cotingidae	*Cephalopterus ornatus*	SCNN1A	NWU14308.1
Aves	Passeriformes	Dicaeidae	*Dicaeum eximium*	SCNN1A	NXH51008.1
Aves	Passeriformes	Dicruridae	*Dicrurus megarhynchus*	SCNN1A	NXJ27103.1
Aves	Passeriformes	Donacobiidae	*Donacobius atricapilla*	SCNN1A	NXB75263.1
Aves	Passeriformes	Elachuridae	*Elachura formosa*	SCNN1A	NXD21605.1
Aves	Passeriformes	Emberizidae	*Emberiza fucata*	SCNN1A	NWR15114.1
Aves	Passeriformes	Estrildidae	*Chloebia gouldiae*	SCNN1A	RLV99042.1
Aves	Passeriformes	Estrildidae	*Taeniopygia guttata*	SCNN1A	XP_002193500.4
Aves	Passeriformes	Eurylaimidae	*Calyptomena viridis*	SCNN1A	NWI50134.1
Aves	Passeriformes	Fringillidae	*Loxia curvirostra*	SCNN1A	NWY91400.1
Aves	Passeriformes	Fringillidae	*Loxia leucoptera*	SCNN1A	NXH02221.1
Aves	Passeriformes	Fringillidae	*Peucedramus taeniatus*	SCNN1A	NXQ09923.1
Aves	Passeriformes	Fringillidae	*Serinus canaria*	SCNN1A	XP_030090837.1
Aves	Passeriformes	Furnariidae	*Furnarius figulus*	SCNN1A	NWR85376.1

Class	Order	Family	Species Name	ENaC subunit	Accession
Aves	Passeriformes	Furnariidae	*Sclerurus mexicanus*	SCNN1A	NXF79461.1
Aves	Passeriformes	Grallariidae	*Grallaria varia*	SCNN1A	NXG17167.1
Aves	Passeriformes	Hirundinidae	*Hirundo rustica*	SCNN1A	XP_039910831.1
Aves	Passeriformes	Icteridae	*Agelaius phoeniceus*	SCNN1A	NWZ04732.1
Aves	Passeriformes	Icteridae	*Quiscalus mexicanus*	SCNN1A	NXQ72869.1
Aves	Passeriformes	Icteridae	*Molothrus ater*	SCNN1A	XP_036240614.1
Aves	Passeriformes	Irenidae	*Chloropsis hardwickii*	SCNN1A	NWH35207.1
Aves	Passeriformes	Irenidae	*Irena cyanogastra*	SCNN1A	NXI10127.1
Aves	Passeriformes	Locustellidae	*Locustella ochotensis*	SCNN1A	NXO44802.1
Aves	Passeriformes	Malaconotidae	*Dryoscopus gambensis*	SCNN1A	NWI79874.1
Aves	Passeriformes	Melanocharitidae	*Oreocharis arfaki*	SCNN1A	NWW01231.1
Aves	Passeriformes	Melanocharitidae	*Cnemophilus loriae*	SCNN1A	NXB10590.1
Aves	Passeriformes	Meliphagidae	*Notiomystis cincta*	SCNN1A	NWX27846.1
Aves	Passeriformes	Meliphagidae	*Struthidea cinerea*	SCNN1A	NXB55468.1
Aves	Passeriformes	Menuridae	*Menura novaehollandiae*	SCNN1A	NXE95198.1
Aves	Passeriformes	Mimidae	*Toxostoma redivivum*	SCNN1A	NWS85360.1
Aves	Passeriformes	Motacillidae	*Motacilla alba*	SCNN1A	NWS08346.1
Aves	Passeriformes	Muscicapidae	*Erithacus rubecula*	SCNN1A	NWY71362.1
Aves	Passeriformes	Muscicapidae	*Cercotrichas coryphoeus*	SCNN1A	NXC83231.1
Aves	Passeriformes	Muscicapidae	*Copsychus sechellarum*	SCNN1A	NXD43953.1
Aves	Passeriformes	Muscicapidae	*Oenanthe oenanthe*	SCNN1A	NXM80841.1
Aves	Passeriformes	Muscicapidae	*Ficedula albicollis*	SCNN1A	XP_005038511.1
Aves	Passeriformes	Nectariniidae	*Leptocoma aspasia*	SCNN1A	NXL82356.1
Aves	Passeriformes	Nicatoridae	*Nicator chloris*	SCNN1A	NXX32576.1
Aves	Passeriformes	Oriolidae	*Oriolus oriolus*	SCNN1A	NXO11773.1
Aves	Passeriformes	Orthonychidae	*Orthonyx spaldingii*	SCNN1A	NXC09617.1
Aves	Passeriformes	Pachycephalidae	*Chaetorhynchus papuensis*	SCNN1A	NXD90565.1
Aves	Passeriformes	Pachycephalidae	*Pachycephala philippinensis*	SCNN1A	NXH99391.1
Aves	Passeriformes	Paradoxornithidae	*Sinosuthora webbiana*	SCNN1A	NWR12182.1
Aves	Passeriformes	Paradoxornithidae	*Brachypodius atriceps*	SCNN1A	NWZ38267.1
Aves	Passeriformes	Pardalotidae	*Pardalotus punctatus*	SCNN1A	NXU10683.1
Aves	Passeriformes	Paridae	*Pseudopodoces humilis*	SCNN1A	XP_005523910.2
Aves	Passeriformes	Paridae	*Cyanistes caeruleus*	SCNN1A	XP_023795998.1
Aves	Passeriformes	Parulidae	*Setophaga kirtlandii*	SCNN1A	NXL24361.1
Aves	Passeriformes	Passerellidae	*Melospiza melodia maxima*	SCNN1A	KAF2981649.1
Aves	Passeriformes	Passerellidae	*Melospiza melodia*	SCNN1A	NWQ54830.1
Aves	Passeriformes	Passerellidae	*Calcarius ornatus*	SCNN1A	NXE61075.1
Aves	Passeriformes	Passerellidae	*Spizella passerina*	SCNN1A	NXX65290.1
Aves	Passeriformes	Passerellidae	*Zonotrichia albicollis*	SCNN1A	XP_005486121.1

Class	Order	Family	Species Name	ENaC subunit	Accession
Aves	Passeriformes	Passeridae	*Passer montanus*	SCNN1A	XP_039573783.1
Aves	Passeriformes	Passeridae	*Pyrgilauda ruficollis*	SCNN1A	XP_041319811.1
Aves	Passeriformes	Pellorneidae	*Illadopsis cleaveri*	SCNN1A	NXM60132.1
Aves	Passeriformes	Petroicidae	*Origma solitaria*	SCNN1A	NWV26461.1
Aves	Passeriformes	Petroicidae	*Edolisoma coerulescens*	SCNN1A	NXH84759.1
Aves	Passeriformes	Petroicidae	*Drymodes brunneopygia*	SCNN1A	NXU36996.1
Aves	Passeriformes	Philepittidae	*Neodrepanis coruscans*	SCNN1A	NXS12199.1
Aves	Passeriformes	Pipridae	*Manacus vitellinus*	SCNN1A	KFW74090.1
Aves	Passeriformes	Pipridae	*Piprites chloris*	SCNN1A	NXK39974.1
Aves	Passeriformes	Pipridae	*Oxyruncus cristatus*	SCNN1A	NXM36863.1
Aves	Passeriformes	Pipridae	*Lepidothrix coronata*	SCNN1A	XP_017670120.1
Aves	Passeriformes	Pipridae	*Corapipo altera*	SCNN1A	XP_027509386.1
Aves	Passeriformes	Pipridae	*Neopelma chrysocephalum*	SCNN1A	XP_027537123.1
Aves	Passeriformes	Pipridae	*Pipra filicauda*	SCNN1A	XP_027586195.1
Aves	Passeriformes	Pipridae	*Chiroxiphia lanceolata*	SCNN1A	XP_032534773.1
Aves	Passeriformes	Pittidae	*Pitta sordida*	SCNN1A	NWI95572.1
Aves	Passeriformes	Platysteiridae	*Platysteira castanea*	SCNN1A	NWU28798.1
Aves	Passeriformes	Polioptilidae	*Polioptila caerulea*	SCNN1A	NWS31428.1
Aves	Passeriformes	Pomatostomidae	*Pomatostomus ruficeps*	SCNN1A	NXS30343.1
Aves	Passeriformes	Prunellidae	*Prunella himalayana*	SCNN1A	NWT70045.1
Aves	Passeriformes	Ptiliogonatidae	*Phainopepla nitens*	SCNN1A	NXO68956.1
Aves	Passeriformes	Ptilonorhynchidae	*Ptilorrhoa leucosticta*	SCNN1A	NXE37449.1
Aves	Passeriformes	Pycnonotidae	*Pycnonotus jocosus*	SCNN1A	NXR73955.1
Aves	Passeriformes	Regulidae	*Regulus satrapa*	SCNN1A	NWR44172.1
Aves	Passeriformes	Remizidae	*Anthoscopus minutus*	SCNN1A	NXQ54749.1
Aves	Passeriformes	Rhabdornithidae	*Rhabdornis inornatus*	SCNN1A	NXH57894.1
Aves	Passeriformes	Rhinocryptidae	*Scytalopus superciliaris*	SCNN1A	NXP27132.1
Aves	Passeriformes	Rhipiduridae	*Rhipidura dahli*	SCNN1A	NXI83294.1
Aves	Passeriformes	Sapayoidae	*Sapayoa aenigma*	SCNN1A	NXA05856.1
Aves	Passeriformes	Sittidae	*Tichodroma muraria*	SCNN1A	NWI07377.1
Aves	Passeriformes	Sittidae	*Sitta europaea*	SCNN1A	NXO78329.1
Aves	Passeriformes	Sturnidae	*Lamprotornis superbus*	SCNN1A	KAG0118240.1
Aves	Passeriformes	Sturnidae	*Leucopsar rothschildi*	SCNN1A	NXB47611.1
Aves	Passeriformes	Sylviidae	*Sylvia atricapilla*	SCNN1A	NWY46574.1
Aves	Passeriformes	Sylviidae	*Eulacestoma nigropectus*	SCNN1A	NXB39457.1
Aves	Passeriformes	Sylviidae	*Sylvia borin*	SCNN1A	NXM93974.1
Aves	Passeriformes	Sylviidae	*Onychostruthus taczanowskii*	SCNN1A	XP_041266859.1
Aves	Passeriformes	Thamnophilidae	*Formicarius rufipectus*	SCNN1A	NXK86059.1
Aves	Passeriformes	Thraupidae	*Nesospiza acunhae*	SCNN1A	NWZ99126.1

Class	Order	Family	Species Name	ENaC subunit	Accession
Aves	Passeriformes	Thraupidae	*Rhodinocichla rosea*	SCNN1A	NXF23647.1
Aves	Passeriformes	Thraupidae	*Geospiza fortis*	SCNN1A	XP_005420651.1
Aves	Passeriformes	Thraupidae	*Camarhynchus parvulus*	SCNN1A	XP_030806070.1
Aves	Passeriformes	Timaliidae	*Grantiella picta*	SCNN1A	NWV34160.1
Aves	Passeriformes	Timaliidae	*Panurus biarmicus*	SCNN1A	NWW39699.1
Aves	Passeriformes	Timaliidae	*Chaetops frenatus*	SCNN1A	NXT72033.1
Aves	Passeriformes	Timaliidae	*Pomatorhinus ruficollis*	SCNN1A	NXY35261.1
Aves	Passeriformes	Tityridae	*Onychorhynchus coronatus*	SCNN1A	NWU75946.1
Aves	Passeriformes	Troglodytidae	*Thryothorus ludovicianus*	SCNN1A	NXA79278.1
Aves	Passeriformes	Turdidae	*Turdus rufiventris*	SCNN1A	KAF4804887.1
Aves	Passeriformes	Turdidae	*Catharus fuscescens*	SCNN1A	NXQ44845.1
Aves	Passeriformes	Tyrannidae	*Neopipo cinnamomea*	SCNN1A	NWQ60918.1
Aves	Passeriformes	Tyrannidae	*Tachuris rubrigastra*	SCNN1A	NWR31684.1
Aves	Passeriformes	Tyrannidae	*Mionectes macconnelli*	SCNN1A	NWT03127.1
Aves	Passeriformes	Tyrannidae	*Tyrannus savana*	SCNN1A	NXM03259.1
Aves	Passeriformes	Tyrannidae	*Empidonax traillii*	SCNN1A	XP_027741048.1
Aves	Passeriformes	Vangidae	*Rhagologus leucostigma*	SCNN1A	NXB26900.1
Aves	Passeriformes	Viduidae	*Vidua chalybeata*	SCNN1A	NXB89315.1
Aves	Passeriformes	Viduidae	*Vidua macroura*	SCNN1A	NXP97228.1
Aves	Passeriformes	Vireonidae	*Vireo altiloquus*	SCNN1A	NWT21158.1
Aves	Passeriformes	Vireonidae	*Sakesphorus luctuosus*	SCNN1A	NXG00712.1
Aves	Passeriformes	Zosteropidae	*Sterrhoptilus dennistouni*	SCNN1A	NXI26858.1
Aves	Passeriformes	Zosteropidae	*Zosterops borbonicus*	SCNN1A	TRZ15383.1
Mammalia	Monotremata	Ornithorhynchidae	*Ornithorhynchus anatinus*	SCNN1A	XP_001512591.3
Mammalia	Monotremata	Tachyglossidae	*Tachyglossus aculeatus*	SCNN1A	XP_038623771.1
Mammalia	Didelphimorphia	Didelphidae	*Marmosa mexicana*	SCNN1A	PRJNA1052681
Mammalia	Dasyuromorphia	Dasyuridae	*Sarcophilus harrisii*	SCNN1A	XP_031794157.1
Mammalia	Diprotodontia	Phascolarctidae	*Phascolarctos cinereus*	SCNN1A	XP_020850002.1
Mammalia	Diprotodontia	Phalangeridae	*Trichosurus vulpecula*	SCNN1A	XP_036616568.1
Mammalia	Diprotodontia	Vombatidae	*Vombatus ursinus*	SCNN1A	XP_027694415.1
Mammalia	Cingulata	Dasypodidae	*Dasypus novemcinctus*	SCNN1A	XP_012384792.1
Mammalia	Pilosa	Megalonychidae	*Choloepus didactylus*	SCNN1A	XP_037701495.1
Mammalia	Sirenia	Trichechidae	*Trichechus manatus latirostris*	SCNN1A	XP_004387318.1
Mammalia	Proboscidea	Elephantidae	*Loxodonta africana*	SCNN1A	XP_003410832.2
Mammalia	Tubulidentata	Orycteropodidae	*Orycteropus afer afer*	SCNN1A	XP_007935387.1
Mammalia	Macroscelidea	Macroscelididae	*Elephantulus edwardii*	SCNN1A	XP_006891420.1
Mammalia	Afrosoricida	Chrysochloridae	*Chrysochloris asiatica*	SCNN1A	XP_006862728.1
Mammalia	Eulipotyphla	Erinaceidae	*Erinaceus europaeus*	SCNN1A	XP_007527906.1
Mammalia	Eulipotyphla	Soricidae	*Sorex araneus*	SCNN1A	XP_004610673.1

Class	Order	Family	Species Name	ENaC subunit	Accession
Mammalia	Eulipotyphla	Talpidae	*Condylura cristata*	SCNN1A	XP_004692925.1
Mammalia	Chiroptera	Hipposideridae	*Hipposideros armiger*	SCNN1A	XP_019483267.1
Mammalia	Chiroptera	Miniopteridae	*Miniopterus natalensis*	SCNN1A	XP_016077262.1
Mammalia	Chiroptera	Molossidae	*Molossus molossus*	SCNN1A	XP_036108337.1
Mammalia	Chiroptera	Phyllostomidae	*Desmodus rotundus*	SCNN1A	XP_024435334.1
Mammalia	Chiroptera	Phyllostomidae	*Phyllostomus discolor*	SCNN1A	XP_028387699.2
Mammalia	Chiroptera	Phyllostomidae	*Sturnira hondurensis*	SCNN1A	XP_036923821.1
Mammalia	Chiroptera	Phyllostomidae	*Artibeus jamaicensis*	SCNN1A	XP_037016038.1
Mammalia	Chiroptera	Pteropodidae	*Rousettus aegyptiacus*	SCNN1A	KAF6497634.1
Mammalia	Chiroptera	Pteropodidae	*Pteropus vampyrus*	SCNN1A	XP_023389772.1
Mammalia	Chiroptera	Pteropodidae	*Pteropus giganteus*	SCNN1A	XP_039730053.1
Mammalia	Chiroptera	Rhinolophidae	*Rhinolophus ferrumequinum*	SCNN1A	KAF6339749.1
Mammalia	Chiroptera	Vespertilionidae	*Myotis brandtii*	SCNN1A	EPQ12362.1
Mammalia	Chiroptera	Vespertilionidae	*Myotis lucifugus*	SCNN1A	XP_006084203.1
Mammalia	Chiroptera	Vespertilionidae	*Myotis davidii*	SCNN1A	XP_006757702.1
Mammalia	Chiroptera	Vespertilionidae	*Eptesicus fuscus*	SCNN1A	XP_028006132.1
Mammalia	Chiroptera	Vespertilionidae	*Myotis myotis*	SCNN1A	XP_036152353.1
Mammalia	Chiroptera	Vespertilionidae	*Pipistrellus kuhlii*	SCNN1A	XP_036311362.1
Mammalia	Pholidota	Manidae	*Manis pentadactyla*	SCNN1A	XP_036765983.1
Mammalia	Pholidota	Manidae	*Manis javanica*	SCNN1A	XP_036864937.1
Mammalia	Carnivora	Canidae	*Nyctereutes procyonoides*	SCNN1A	CAD7670422.1
Mammalia	Carnivora	Canidae	*Canis lupus familiaris*	SCNN1A	XP_005637311.1
Mammalia	Carnivora	Canidae	*Canis lupus dingo*	SCNN1A	XP_025317715.2
Mammalia	Carnivora	Canidae	*Vulpes vulpes*	SCNN1A	XP_025851151.1
Mammalia	Carnivora	Felidae	*Lynx pardinus*	SCNN1A	VFV18502.1
Mammalia	Carnivora	Felidae	*Panthera tigris altaica*	SCNN1A	XP_015396310.1
Mammalia	Carnivora	Felidae	*Panthera pardus*	SCNN1A	XP_019320300.1
Mammalia	Carnivora	Felidae	*Puma concolor*	SCNN1A	XP_025786014.1
Mammalia	Carnivora	Felidae	*Acinonyx jubatus*	SCNN1A	XP_026929903.1
Mammalia	Carnivora	Felidae	*Lynx canadensis*	SCNN1A	XP_030177357.1
Mammalia	Carnivora	Herpestidae	*Suricata suricatta*	SCNN1A	XP_029809617.1
Mammalia	Carnivora	Hyaenidae	*Crocuta crocuta*	SCNN1A	KAF0882719.1
Mammalia	Carnivora	Mustelidae	*Mustela putorius furo*	SCNN1A	XP_004766774.1
Mammalia	Carnivora	Mustelidae	*Enhydra lutris kenyoni*	SCNN1A	XP_022378086.1
Mammalia	Carnivora	Mustelidae	*Lontra canadensis*	SCNN1A	XP_032730688.1
Mammalia	Carnivora	Otariidae	*Callorhinus ursinus*	SCNN1A	XP_025707522.1
Mammalia	Carnivora	Otariidae	*Zalophus californianus*	SCNN1A	XP_027449566.1
Mammalia	Carnivora	Otariidae	*Eumetopias jubatus*	SCNN1A	XP_027976909.1
Mammalia	Carnivora	Phocidae	*Neomonachus schauinslandi*	SCNN1A	XP_021546481.1

Class	Order	Family	Species Name	ENaC subunit	Accession
Mammalia	Carnivora	Phocidae	*Phoca vitulina*	SCNN1A	XP_032246935.1
Mammalia	Carnivora	Phocidae	*Mirounga leonina*	SCNN1A	XP_034884292.1
Mammalia	Carnivora	Phocidae	*Halichoerus grypus*	SCNN1A	XP_035973530.1
Mammalia	Carnivora	Ursidae	*Ursus arctos horribilis*	SCNN1A	XP_026358400.1
Mammalia	Carnivora	Ursidae	*Ailuropoda melanoleuca*	SCNN1A	XP_034500988.1
Mammalia	Perissodactyla	Equidae	*Equus przewalskii*	SCNN1A	XP_008512147.1
Mammalia	Perissodactyla	Equidae	*Equus asinus*	SCNN1A	XP_014697210.1
Mammalia	Perissodactyla	Equidae	*Equus caballus*	SCNN1A	XP_023498785.1
Mammalia	Perissodactyla	Rhinocerotidae	*Diceros bicornis minor*	SCNN1A	KAF5912361.1
Mammalia	Perissodactyla	Rhinocerotidae	*Ceratotherium simum simum*	SCNN1A	XP_004438792.2
Mammalia	Artiodactyla	Bovidae	*Ovis aries*	SCNN1A	KAG5212611.1
Mammalia	Artiodactyla	Bovidae	*Bos mutus*	SCNN1A	MXQ98601.1
Mammalia	Artiodactyla	Bovidae	*Bubalus bubalis*	SCNN1A	XP_006065845.1
Mammalia	Artiodactyla	Bovidae	*Bison bison bison*	SCNN1A	XP_010843500.1
Mammalia	Artiodactyla	Bovidae	*Capra hircus*	SCNN1A	XP_017904088.1
Mammalia	Artiodactyla	Bovidae	*Bos taurus*	SCNN1A	XP_024847138.1
Mammalia	Artiodactyla	Bovidae	*Oryx dammah*	SCNN1A	XP_040090886.1
Mammalia	Artiodactyla	Camelidae	*Camelus dromedarius*	SCNN1A	XP_031300111.1
Mammalia	Artiodactyla	Camelidae	*Camelus ferus*	SCNN1A	XP_032328764.1
Mammalia	Artiodactyla	Cervidae	*Cervus elaphus hippelaphus*	SCNN1A	OWK04048.1
Mammalia	Artiodactyla	Cervidae	*Muntiacus muntjak*	SCNN1A	KAB0353011.1
Mammalia	Artiodactyla	Cervidae	*Cervus hanglu yarkandensis*	SCNN1A	KAF4022875.1
Mammalia	Artiodactyla	Cervidae	*Odocoileus virginianus texanus*	SCNN1A	XP_020734323.1
Mammalia	Artiodactyla	Suidae	*Sus scrofa*	SCNN1A	XP_020946765.1
Mammalia	Artiodactyla	Balaenopteridae	*Balaenoptera physalus*	SCNN1A	KAB0395735.1
Mammalia	Artiodactyla	Balaenopteridae	*Balaenoptera acutorostrata scammoni*	SCNN1A	XP_007170321.1
Mammalia	Artiodactyla	Balaenopteridae	*Balaenoptera musculus*	SCNN1A	XP_036722300.1
Mammalia	Artiodactyla	Delphinidae	*Sousa chinensis*	SCNN1A	TEA30503.1
Mammalia	Artiodactyla	Delphinidae	*Lagenorhynchus obliquidens*	SCNN1A	XP_026949156.1
Mammalia	Artiodactyla	Delphinidae	*Globicephala melas*	SCNN1A	XP_030690515.1
Mammalia	Artiodactyla	Delphinidae	*Orcinus orca*	SCNN1A	XP_033260180.1
Mammalia	Artiodactyla	Monodontidae	*Monodon monoceros*	SCNN1A	TKC52160.1
Mammalia	Artiodactyla	Monodontidae	*Delphinapterus leucas*	SCNN1A	XP_022433075.1
Mammalia	Artiodactyla	Phocoenidae	*Neophocaena asiaeorientalis asiaeorientalis*	SCNN1A	XP_024589219.1
Mammalia	Artiodactyla	Physeteridae	*Physeter catodon*	SCNN1A	XP_028346744.1
Mammalia	Scandentia	Tupaiidae	*Tupaia chinensis*	SCNN1A	XP_027625032.1
Mammalia	Lagomorpha	Leporidae	*Oryctolagus cuniculus*	SCNN1A	AAS00455.1
Mammalia	Lagomorpha	Leporidae	*Lepus yarkandensis*	SCNN1A	QGN65848.1
Mammalia	Lagomorpha	Ochotonidae	*Ochotona princeps*	SCNN1A	XP_004596510.1

Class	Order	Family	Species Name	ENaC subunit	Accession
Mammalia	Rodentia	Bathyergidae	*Fukomys damarensis*	SCNN1A	KFO20244.1
Mammalia	Rodentia	Bathyergidae	*Heterocephalus glaber*	SCNN1A	XP_012922790.1
Mammalia	Rodentia	Caviidae	*Cavia porcellus*	SCNN1A	CAB64910.1
Mammalia	Rodentia	Chinchillidae	*Chinchilla lanigera*	SCNN1A	XP_005378822.1
Mammalia	Rodentia	Cricetidae	*Peromyscus maniculatus bairdii*	SCNN1A	XP_006992162.2
Mammalia	Rodentia	Cricetidae	*Cricetulus griseus*	SCNN1A	XP_027284123.1
Mammalia	Rodentia	Cricetidae	*Peromyscus leucopus*	SCNN1A	XP_028748183.1
Mammalia	Rodentia	Cricetidae	*Onychomys torridus*	SCNN1A	XP_036038466.1
Mammalia	Rodentia	Cricetidae	*Arvicola amphibius*	SCNN1A	XP_038175880.1
Mammalia	Rodentia	Cricetidae	*Mesocricetus auratus*	SCNN1A	XP_040595358.1
Mammalia	Rodentia	Dipodidae	*Jaculus jaculus*	SCNN1A	XP_012806664.1
Mammalia	Rodentia	Echinopsidae	*Echinops telfairi*	SCNN1A	XP_004708279.2
Mammalia	Rodentia	Heteromyidae	*Dipodomys ordii*	SCNN1A	XP_012879047.1
Mammalia	Rodentia	Muridae	*Mus musculus*	SCNN1A	NP_035454.2
Mammalia	Rodentia	Muridae	*Rattus norvegicus*	SCNN1A	NP_113736.1
Mammalia	Rodentia	Muridae	*Mus pahari*	SCNN1A	XP_021046590.1
Mammalia	Rodentia	Muridae	*Meriones unguiculatus*	SCNN1A	XP_021492615.1
Mammalia	Rodentia	Muridae	*Mastomys coucha*	SCNN1A	XP_031239318.1
Mammalia	Rodentia	Muridae	*Arvicanthis niloticus*	SCNN1A	XP_034367600.1
Mammalia	Rodentia	Octodontidae	*Octodon degus*	SCNN1A	XP_023559504.1
Mammalia	Rodentia	Sciuridae	*Marmota monax*	SCNN1A	VTJ64549.1
Mammalia	Rodentia	Sciuridae	*Ictidomys tridecemlineatus*	SCNN1A	XP_005338417.1
Mammalia	Rodentia	Sciuridae	*Marmota marmota marmota*	SCNN1A	XP_015356212.1
Mammalia	Rodentia	Sciuridae	*Marmota flaviventris*	SCNN1A	XP_027806870.1
Mammalia	Rodentia	Spalacidae	*Nannospalax galili*	SCNN1A	XP_008823654.2
Mammalia	Dermoptera	Cynocephalidae	*Galeopterus variegatus*	SCNN1A	XP_008588158.1
Mammalia	Primates	Aotidae	*Aotus nancymaae*	SCNN1A	XP_012311349.1
Mammalia	Primates	Callitrichidae	*Callithrix jacchus*	SCNN1A	XP_035111378.1
Mammalia	Primates	Cebidae	*Cebus imitator*	SCNN1A	XP_017401644.1
Mammalia	Primates	Cebidae	*Sapajus apella*	SCNN1A	XP_032110434.1
Mammalia	Primates	Cebidae	*Saimiri boliviensis boliviensis*	SCNN1A	XP_039317568.1
Mammalia	Primates	Cercopithecidae	*Macaca fascicularis*	SCNN1A	XP_005569956.1
Mammalia	Primates	Cercopithecidae	*Chlorocebus sabaeus*	SCNN1A	XP_007965522.2
Mammalia	Primates	Cercopithecidae	*Rhinopithecus roxellana*	SCNN1A	XP_010384263.1
Mammalia	Primates	Cercopithecidae	*Macaca nemestrina*	SCNN1A	XP_011743854.1
Mammalia	Primates	Cercopithecidae	*Colobus angolensis palliatus*	SCNN1A	XP_011807118.1
Mammalia	Primates	Cercopithecidae	*Mandrillus leucophaeus*	SCNN1A	XP_011838331.1
Mammalia	Primates	Cercopithecidae	*Cercocebus atys*	SCNN1A	XP_011909850.1
Mammalia	Primates	Cercopithecidae	*Macaca mulatta*	SCNN1A	XP_015006375.2

Class	Order	Family	Species Name	ENaC subunit	Accession
Mammalia	Primates	Cercopithecidae	*Rhinopithecus bieti*	SCNN1A	XP_017738774.1
Mammalia	Primates	Cercopithecidae	*Papio anubis*	SCNN1A	XP_021777631.1
Mammalia	Primates	Cercopithecidae	*Theropithecus gelada*	SCNN1A	XP_025258766.1
Mammalia	Primates	Cercopithecidae	*Trachypithecus francoisi*	SCNN1A	XP_033079005.1
Mammalia	Primates	Cheirogaleidae	*Microcebus murinus*	SCNN1A	XP_020143274.1
Mammalia	Primates	Galagonidae	*Otolemur garnettii*	SCNN1A	XP_003796461.2
Mammalia	Primates	Hominidae	*Homo sapiens*	SCNN1A	NP_001029.1
Mammalia	Primates	Hominidae	*Pongo abelii*	SCNN1A	PNJ30215.1
Mammalia	Primates	Hominidae	*Pan paniscus*	SCNN1A	XP_003820365.2
Mammalia	Primates	Hominidae	*Gorilla gorilla gorilla*	SCNN1A	XP_018894070.1
Mammalia	Primates	Hominidae	*Pan troglodytes*	SCNN1A	XP_024203415.1
Mammalia	Primates	Hylobatidae	*Nomascus leucogenys*	SCNN1A	XP_030660590.1
Mammalia	Primates	Hylobatidae	*Hylobates moloch*	SCNN1A	XP_032015265.1
Mammalia	Primates	Indriidae	*Propithecus coquereli*	SCNN1A	XP_012493336.1
Sarcopterygii	Coelacanthiformes	Latimeriidae	*Latimeria chalumnae*	SCNN1D	XP_005986143.1
Amphibia	Gymnophiona	Dermophiidae	*Geotrypetes seraphini*	SCNN1D	XP_033778426.1
Amphibia	Gymnophiona	Siphonopidae	*Microcaecilia unicolor*	SCNN1D	XP_030078006.1
Amphibia	Anura	Bufonidae	*Bufo bufo*	SCNN1D	XP_040276107.1
Amphibia	Anura	Dicroglossidae	*Nanorana parkeri*	SCNN1D	XP_018424177.1
Amphibia	Anura	Pipidae	*Xenopus laevis*	SCNN1D	NP_001082645.1
Amphibia	Anura	Pipidae	*Xenopus tropicalis*	SCNN1D	XP_031762206.1
Amphibia	Anura	Ranidae	*Rana temporaria*	SCNN1D	XP_040181776.1
Reptilia	Squamata	Agamidae	*Pogona vitticeps*	SCNN1D	XP_020637654.1
Reptilia	Squamata	Colubridae	*Thamnophis elegans*	SCNN1D	XP_032088084.1
Reptilia	Squamata	Colubridae	*Pantherophis guttatus*	SCNN1D	XP_034290791.1
Reptilia	Squamata	Dactyloidae	*Anolis carolinensis*	SCNN1D	XP_062822039.1
Reptilia	Squamata	Elapidae	*Pseudonaja textilis*	SCNN1D	XP_026571554.1
Reptilia	Squamata	Gekkonidae	*Paroedura picta*	SCNN1D	GCF49088.1
Reptilia	Squamata	Lacertidae	*Podarcis muralis*	SCNN1D	XP_028596662.1
Reptilia	Squamata	Lacertidae	*Lacerta agilis*	SCNN1D	XP_033012199.1
Reptilia	Squamata	Lacertidae	*Zootoca vivipara*	SCNN1D	XP_034975937.1
Reptilia	Squamata	Pythonidae	*Python bivittatus*	SCNN1D	XP_025021199.1
Reptilia	Squamata	Viperidae	*Protobothrops mucrosquamatus*	SCNN1D	XP_015671537.1
Reptilia	Squamata	Viperidae	*Crotalus tigris*	SCNN1D	XP_039223167.1
Reptilia	Testudines	Cheloniidae	*Chelonia mydas*	SCNN1D	XP_027688197.2
Reptilia	Testudines	Emydidae	*Terrapene carolina triunguis*	SCNN1D	XP_026515859.1
Reptilia	Testudines	Emydidae	*Trachemys scripta elegans*	SCNN1D	XP_034609336.1
Reptilia	Testudines	Geoemydidae	*Mauremys reevesii*	SCNN1D	XP_039365297.1
Reptilia	Testudines	Kinosternidae	*Platysternon megacephalum*	SCNN1D	TFK13677.1

Class	Order	Family	Species Name	ENaC subunit	Accession
Reptilia	Testudines	Testudinidae	*Gopherus evgoodei*	SCNN1D	XP_030393490.1
Reptilia	Testudines	Testudinidae	*Chelonoidis abingdonii*	SCNN1D	XP_032635837.1
Reptilia	Testudines	Trionychidae	*Pelodiscus sinensis*	SCNN1D	XP_014431009.1
Reptilia	Crocodilia	Alligatoridae	*Alligator mississippiensis*	SCNN1D	XP_019349363.1
Reptilia	Crocodilia	Alligatoridae	*Alligator sinensis*	SCNN1D	XP_025053925.1
Reptilia	Crocodilia	Crocodylidae	*Crocodylus porosus*	SCNN1D	XP_019397131.1
Reptilia	Crocodilia	Gavialidae	*Gavialis gangeticus*	SCNN1D	XP_019367370.1
Aves	Struthioniformes	Struthionidae	*Struthio camelus australis*	SCNN1D	XP_009669810.1
Aves	Tinamiformes	Tinamidae	*Crypturellus soui*	SCNN1D	NWI20306.1
Aves	Tinamiformes	Tinamidae	*Nothoprocta pentlandii*	SCNN1D	NWX88720.1
Aves	Tinamiformes	Tinamidae	*Tinamus guttatus*	SCNN1D	XP_010210790.1
Aves	Apterygiformes	Apterygidae	*Apteryx mantelli mantelli*	SCNN1D	XP_013811509.1
Aves	Casuariiformes	Dromaiidae	*Dromaius novaehollandiae*	SCNN1D	XP_025956438.1
Aves	Anseriformes	Anatidae	*Asarcornis scutulata*	SCNN1D	NWZ23027.1
Aves	Anseriformes	Anatidae	*Anas platyrhynchos*	SCNN1D	XP_005008876.2
Aves	Anseriformes	Anatidae	*Aythya fuligula*	SCNN1D	XP_032057175.1
Aves	Anseriformes	Anatidae	*Oxyura jamaicensis*	SCNN1D	XP_035200520.1
Aves	Anseriformes	Anhimidae	*Chauna torquata*	SCNN1D	NXK56546.1
Aves	Anseriformes	Anseranatidae	*Anseranas semipalmata*	SCNN1D	NXI71067.1
Aves	Galliformes	Cracidae	*Nothocercus julius*	SCNN1D	NXA51988.1
Aves	Galliformes	Cracidae	*Penelope pileata*	SCNN1D	NXC46646.1
Aves	Galliformes	Cracidae	*Nothocercus nigrocapillus*	SCNN1D	NXD10910.1
Aves	Galliformes	Megapodiidae	*Alectura lathami*	SCNN1D	NXL95324.1
Aves	Galliformes	Numididae	*Numida meleagris*	SCNN1D	XP_021230336.1
Aves	Galliformes	Odontophoridae	*Odontophorus gujanensis*	SCNN1D	NXJ05299.1
Aves	Galliformes	Odontophoridae	*Callipepla squamata*	SCNN1D	OXB60992.1
Aves	Galliformes	Odontophoridae	*Colinus virginianus*	SCNN1D	OXB78169.1
Aves	Galliformes	Phasianidae	*Lophotis ruficrista*	SCNN1D	NXE15550.1
Aves	Galliformes	Phasianidae	*Bambusicola thoracicus*	SCNN1D	POI33736.1
Aves	Galliformes	Phasianidae	*Coturnix japonica*	SCNN1D	XP_032304611.1
Aves	Galliformes	Phasianidae	*Gallus gallus*	SCNN1D	XP_004947475.2
Aves	Phoenicopteriformes	Phoenicopteridae	*Phoenicopterus ruber ruber*	SCNN1D	KFQ85501.1
Aves	Podicipediformes	Podicipedidae	*Podiceps cristatus*	SCNN1D	KFZ57524.1
Aves	Podicipediformes	Podicipedidae	*Podilymbus podiceps*	SCNN1D	NXL46643.1
Aves	Columbiformes	Columbidae	*Columbina picui*	SCNN1D	NWQ82484.1
Aves	Columbiformes	Columbidae	*Caloenas nicobarica*	SCNN1D	NWX04113.1
Aves	Columbiformes	Columbidae	*Patagioenas fasciata monilis*	SCNN1D	OPJ86087.1
Aves	Columbiformes	Columbidae	*Columba livia*	SCNN1D	XP_021137584.1
Aves	Pterocliformes	Pteroclidae	*Pterocles gutturalis*	SCNN1D	KFV05873.1

Class	Order	Family	Species Name	ENaC subunit	Accession
Aves	Mesitornithiformes	Mesitornithidae	*Mesitornis unicolor*	SCNN1D	XP_010185680.1
Aves	Cuculiformes	Cuculidae	*Geococcyx californianus*	SCNN1D	NWH66695.1
Aves	Cuculiformes	Cuculidae	*Crotophaga sulcirostris*	SCNN1D	NWS69582.1
Aves	Cuculiformes	Cuculidae	*Rhinoptilus africanus*	SCNN1D	NXN40788.1
Aves	Cuculiformes	Cuculidae	*Cuculus canorus*	SCNN1D	XP_009555057.1
Aves	Otidiformes	Otididae	*Ardeotis kori*	SCNN1D	NXE20016.1
Aves	Musophagiformes	Musophagidae	*Tauraco erythrolophus*	SCNN1D	XP_009988730.1
Aves	Caprimulgiformes	Aegothelidae	*Aegotheles bennettii*	SCNN1D	NWX22426.1
Aves	Caprimulgiformes	Caprimulgidae	*Chordeiles acutipennis*	SCNN1D	NXL57152.1
Aves	Caprimulgiformes	Caprimulgidae	*Nyctiprogne leucopyga*	SCNN1D	NXW43319.1
Aves	Caprimulgiformes	Caprimulgidae	*Antrostomus carolinensis*	SCNN1D	XP_010175753.1
Aves	Caprimulgiformes	Nyctibiidae	*Nyctibius bracteatus*	SCNN1D	NXF30417.1
Aves	Caprimulgiformes	Nyctibiidae	*Nyctibius grandis*	SCNN1D	NXQ81407.1
Aves	Caprimulgiformes	Podargidae	*Podargus strigoides*	SCNN1D	NXX15905.1
Aves	Caprimulgiformes	Steatornithidae	*Steatornis caripensis*	SCNN1D	NWX47620.1
Aves	Opisthocomiformes	Opisthocomidae	*Opisthocomus hoazin*	SCNN1D	XP_009940173.1
Aves	Gruiformes	Aramidae	*Aramus guarauna*	SCNN1D	NXO46941.1
Aves	Gruiformes	Cariamidae	*Cariama cristata*	SCNN1D	XP_009696851.1
Aves	Gruiformes	Eurypygidae	*Eurypyga helias*	SCNN1D	XP_010156624.1
Aves	Gruiformes	Gruidae	*Balearica regulorum gibbericeps*	SCNN1D	XP_010298429.1
Aves	Gruiformes	Heliornithidae	*Heliornis fulica*	SCNN1D	NXP44749.1
Aves	Gruiformes	Psophiidae	*Psophia crepitans*	SCNN1D	NXI90373.1
Aves	Gruiformes	Rallidae	*Zapornia atra*	SCNN1D	NXT81529.1
Aves	Gruiformes	Rallidae	*Nipponia nippon*	SCNN1D	XP_009459285.1
Aves	Gruiformes	Rhynochetidae	*Rhynochetos jubatus*	SCNN1D	NWW88250.1
Aves	Charadriiformes	Alcidae	*Alca torda*	SCNN1D	NWX69072.1
Aves	Charadriiformes	Alcidae	*Uria aalge*	SCNN1D	NXV51397.1
Aves	Charadriiformes	Burhinidae	*Burhinus bistriatus*	SCNN1D	NWQ93322.1
Aves	Charadriiformes	Charadriidae	*Eudromia elegans*	SCNN1D	NXA40278.1
Aves	Charadriiformes	Charadriidae	*Charadrius vociferus*	SCNN1D	XP_009887967.1
Aves	Charadriiformes	Chionidae	*Chionis minor*	SCNN1D	NWY49930.1
Aves	Charadriiformes	Dromadidae	*Dromas ardeola*	SCNN1D	NWU48592.1
Aves	Charadriiformes	Glareolidae	*Glareola pratincola*	SCNN1D	NXY72737.1
Aves	Charadriiformes	Jacanidae	*Jacana jacana*	SCNN1D	NXS93465.1
Aves	Charadriiformes	Laridae	*Chroicocephalus maculipennis*	SCNN1D	NWT41978.1
Aves	Charadriiformes	Laridae	*Rynchops niger*	SCNN1D	NXN50236.1
Aves	Charadriiformes	Laridae	*Rissa tridactyla*	SCNN1D	NXV35382.1
Aves	Charadriiformes	Otididae	*Chlamydotis macqueenii*	SCNN1D	XP_010120102.1
Aves	Charadriiformes	Pluvianellidae	*Pluvianellus socialis*	SCNN1D	NXT58126.1

Class	Order	Family	Species Name	ENaC subunit	Accession
Aves	Charadriiformes	Recurvirostridae	*Himantopus himantopus*	SCNN1D	NXN76312.1
Aves	Charadriiformes	Rostratulidae	*Rostratula benghalensis*	SCNN1D	NXJ64910.1
Aves	Charadriiformes	Scolopacidae	*Arenaria interpres*	SCNN1D	NXK16322.1
Aves	Charadriiformes	Scolopacidae	*Syrrhaptes paradoxus*	SCNN1D	NXT17992.1
Aves	Charadriiformes	Scolopacidae	*Calidris pugnax*	SCNN1D	XP_014796825.1
Aves	Charadriiformes	Stercorariidae	*Stercorarius parasiticus*	SCNN1D	NXG85551.1
Aves	Charadriiformes	Thinocoridae	*Thinocorus orbignyianus*	SCNN1D	NXP14533.1
Aves	Charadriiformes	Turnicidae	*Turnix velox*	SCNN1D	NXU47721.1
Aves	Apodiformes	Apodidae	*Chaetura pelagica*	SCNN1D	KFU95381.1
Aves	Apodiformes	Trochilidae	*Oreotrochilus melanogaster*	SCNN1D	NXU76082.1
Aves	Apodiformes	Trochilidae	*Calypte anna*	SCNN1D	XP_008501928.2
Aves	Gaviiformes	Gaviidae	*Gavia stellata*	SCNN1D	KFV47250.1
Aves	Sphenisciformes	Spheniscidae	*Spheniscus magellanicus*	SCNN1D	KAF1399825.1
Aves	Sphenisciformes	Spheniscidae	*Pygoscelis papua*	SCNN1D	KAF1442053.1
Aves	Sphenisciformes	Spheniscidae	*Eudyptula novaehollandiae*	SCNN1D	KAF1474385.1
Aves	Sphenisciformes	Spheniscidae	*Eudyptula albosignata*	SCNN1D	KAF1533217.1
Aves	Sphenisciformes	Spheniscidae	*Eudyptes robustus*	SCNN1D	KAF1657190.1
Aves	Sphenisciformes	Spheniscidae	*Aptenodytes patagonicus*	SCNN1D	KAF1661705.1
Aves	Sphenisciformes	Spheniscidae	*Aptenodytes forsteri*	SCNN1D	XP_009272513.1
Aves	Sphenisciformes	Spheniscidae	*Pygoscelis adeliae*	SCNN1D	XP_009323536.1
Aves	Ciconiiformes	Ciconiidae	*Ciconia maguari*	SCNN1D	NXJ37922.1
Aves	Suliformes	Anhingidae	*Anhinga anhinga*	SCNN1D	NXC69615.1
Aves	Suliformes	Anhingidae	*Anhinga rufa*	SCNN1D	NXT88645.1
Aves	Suliformes	Fregatidae	*Fregata magnificens*	SCNN1D	NWH51152.1
Aves	Suliformes	Phalacrocoracidae	*Phalacrocorax carbo*	SCNN1D	XP_009509950.1
Aves	Suliformes	Sulidae	*Sula dactylatra*	SCNN1D	NWI29419.1
Aves	Procellariiformes	Diomedeidae	*Thalassarche chlororhynchos*	SCNN1D	NXU28145.1
Aves	Procellariiformes	Hydrobatidae	*Oceanodroma tethys*	SCNN1D	NXH77130.1
Aves	Procellariiformes	Oceanitidae	*Fregetta grallaria*	SCNN1D	NXW05858.1
Aves	Procellariiformes	Pelecanoididae	*Pelecanoides urinatrix*	SCNN1D	NXT42957.1
Aves	Procellariiformes	Procellariidae	*Calonectris borealis*	SCNN1D	NXV87018.1
Aves	Procellariiformes	Procellariidae	*Fulmarus glacialis*	SCNN1D	XP_009578785.1
Aves	Pelecaniformes	Ardeidae	*Cochlearius cochlearius*	SCNN1D	NXE80541.1
Aves	Pelecaniformes	Ardeidae	*Egretta garzetta*	SCNN1D	XP_009645087.1
Aves	Pelecaniformes	Balaenicipitidae	*Balaeniceps rex*	SCNN1D	NXS47091.1
Aves	Pelecaniformes	Pelecanidae	*Pelecanus crispus*	SCNN1D	XP_009481490.1
Aves	Pelecaniformes	Phaethontidae	*Phaetusa simplex*	SCNN1D	NXW37782.1
Aves	Pelecaniformes	Threskiornithidae	*Ibidorhyncha struthersii*	SCNN1D	NXA24214.1
Aves	Pelecaniformes	Threskiornithidae	*Mesembrinibis cayennensis*	SCNN1D	NXL12165.1

Class	Order	Family	Species Name	ENaC subunit	Accession
Aves	Strigiformes	Strigidae	*Ciccaba nigrolineata*	SCNN1D	NXF63850.1
Aves	Strigiformes	Strigidae	*Glaucidium brasilianum*	SCNN1D	NXL27695.1
Aves	Strigiformes	Strigidae	*Athene cunicularia*	SCNN1D	XP_026718565.1
Aves	Strigiformes	Tytonidae	*Tyto alba alba*	SCNN1D	XP_032863374.1
Aves	Accipitriformes	Accipitridae	*Spizaetus tyrannus*	SCNN1D	NXJ48698.1
Aves	Accipitriformes	Accipitridae	*Circaetus pectoralis*	SCNN1D	NXW13522.1
Aves	Accipitriformes	Accipitridae	*Haliaeetus albicilla*	SCNN1D	XP_009910145.1
Aves	Accipitriformes	Accipitridae	*Haliaeetus leucocephalus*	SCNN1D	XP_010575842.1
Aves	Accipitriformes	Accipitridae	*Aquila chrysaetos chrysaetos*	SCNN1D	XP_029875133.1
Aves	Accipitriformes	Cathartidae	*Cathartes aura*	SCNN1D	KFP50332.1
Aves	Accipitriformes	Pandionidae	*Pandion haliaetus*	SCNN1D	NXS75091.1
Aves	Accipitriformes	Sagittariidae	*Sagittarius serpentarius*	SCNN1D	NXQ94816.1
Aves	Coliiformes	Coliidae	*Colius striatus*	SCNN1D	KFP29097.1
Aves	Coliiformes	Coliidae	*Urocolius indicus*	SCNN1D	NXX75109.1
Aves	Trogoniformes	Trogonidae	*Trogon melanurus*	SCNN1D	NXJ82532.1
Aves	Bucerotiformes	Bucerotidae	*Buceros rhinoceros silvestris*	SCNN1D	XP_010135402.1
Aves	Bucerotiformes	Bucorvidae	*Bucorvus abyssinicus*	SCNN1D	NWR59579.1
Aves	Bucerotiformes	Phoeniculidae	*Apaloderma vittatum*	SCNN1D	XP_009868858.1
Aves	Bucerotiformes	Upupidae	*Upupa epops*	SCNN1D	NWU91471.1
Aves	Piciformes	Bucconidae	*Bucco capensis*	SCNN1D	NXH12505.1
Aves	Piciformes	Capitonidae	*Eubucco bourcierii*	SCNN1D	NXF95656.1
Aves	Piciformes	Galbulidae	*Baryphthengus martii*	SCNN1D	NXG80832.1
Aves	Piciformes	Galbulidae	*Galbula dea*	SCNN1D	NXI40105.1
Aves	Piciformes	Indicatoridae	*Indicator maculatus*	SCNN1D	NXN18134.1
Aves	Piciformes	Lybiidae	*Tricholaema leucomelas*	SCNN1D	NXX50580.1
Aves	Piciformes	Megalaimidae	*Psilopogon haemacephalus*	SCNN1D	NXG45394.1
Aves	Piciformes	Megalaimidae	*Serilophus lunatus*	SCNN1D	NXM76250.1
Aves	Piciformes	Picidae	*Dryobates pubescens*	SCNN1D	KFV72326.1
Aves	Piciformes	Ramphastidae	*Ramphastos sulfuratus*	SCNN1D	NXP81238.1
Aves	Coraciiformes	Alcedinidae	*Halcyon senegalensis*	SCNN1D	NXD79678.1
Aves	Coraciiformes	Alcedinidae	*Chloroceryle aenea*	SCNN1D	NXI58284.1
Aves	Coraciiformes	Alcedinidae	*Eurystomus gularis*	SCNN1D	NXW61853.1
Aves	Coraciiformes	Alcedinidae	*Ceyx cyanopectus*	SCNN1D	NXY91382.1
Aves	Coraciiformes	Brachypteraciidae	*Brachypteracias leptosomus*	SCNN1D	NXS54209.1
Aves	Coraciiformes	Leptosomidae	*Leptosomus discolor*	SCNN1D	KFQ14849.1
Aves	Coraciiformes	Phoeniculidae	*Rhinopomastus cyanomelas*	SCNN1D	NXN98538.1
Aves	Coraciiformes	Todidae	*Todus mexicanus*	SCNN1D	NWI63147.1
Aves	Falconiformes	Falconidae	*Falco peregrinus*	SCNN1D	XP_005238076.1
Aves	Falconiformes	Falconidae	*Falco rusticolus*	SCNN1D	XP_037236674.1

Class	Order	Family	Species Name	ENaC subunit	Accession
Aves	Falconiformes	Falconidae	*Falco naumanni*	SCNN1D	XP_040443157.1
Aves	Psittaciformes	Cacatuidae	*Probosciger aterrimus*	SCNN1D	NWS43684.1
Aves	Psittaciformes	Psittacidae	*Amazona guildingii*	SCNN1D	NXK81785.1
Aves	Psittaciformes	Psittacidae	*Melopsittacus undulatus*	SCNN1D	XP_005143759.2
Aves	Psittaciformes	Psittaculidae	*Eolophus roseicapilla*	SCNN1D	NXD65719.1
Aves	Psittaciformes	Strigopidae	*Nestor notabilis*	SCNN1D	KFQ46795.1
Aves	Psittaciformes	Strigopidae	*Strigops habroptila*	SCNN1D	XP_030363702.1
Aves	Passeriformes	Acanthizidae	*Dasyornis broadbenti*	SCNN1D	NWV77641.1
Aves	Passeriformes	Alaudidae	*Alaudala cheleensis*	SCNN1D	NXQ26196.1
Aves	Passeriformes	Artamidae	*Corythaeola cristata*	SCNN1D	NXC12166.1
Aves	Passeriformes	Artamidae	*Gymnorhina tibicen*	SCNN1D	NXM41369.1
Aves	Passeriformes	Bombycillidae	*Urocynchramus pylzowi*	SCNN1D	NWU01473.1
Aves	Passeriformes	Campephagidae	*Brachypodius atriceps*	SCNN1D	NWZ43822.1
Aves	Passeriformes	Campephagidae	*Edolisoma coerulescens*	SCNN1D	NXH85680.1
Aves	Passeriformes	Cardinalidae	*Cardinalis cardinalis*	SCNN1D	NWT30580.1
Aves	Passeriformes	Cardinalidae	*Pheucticus melanocephalus*	SCNN1D	NWY35867.1
Aves	Passeriformes	Cinclosomatidae	*Pedionomus torquatus*	SCNN1D	NWW51359.1
Aves	Passeriformes	Cisticolidae	*Cisticola juncidis*	SCNN1D	NXO21296.1
Aves	Passeriformes	Climacteridae	*Climacteris rufus*	SCNN1D	NWW74589.1
Aves	Passeriformes	Corcoracidae	*Struthidea cinerea*	SCNN1D	NXB60724.1
Aves	Passeriformes	Corvidae	*Aphelocoma coerulescens*	SCNN1D	NWY17777.1
Aves	Passeriformes	Corvidae	*Corvus brachyrhynchos*	SCNN1D	XP_017599463.1
Aves	Passeriformes	Corvidae	*Corvus cornix cornix*	SCNN1D	XP_019145331.2
Aves	Passeriformes	Corvidae	*Corvus moneduloides*	SCNN1D	XP_031987350.1
Aves	Passeriformes	Cotingidae	*Cephalopterus ornatus*	SCNN1D	NWU11556.1
Aves	Passeriformes	Dasyornithidae	*Falcunculus frontatus*	SCNN1D	NWW18378.1
Aves	Passeriformes	Dicruridae	*Dicrurus megarhynchus*	SCNN1D	NXJ25911.1
Aves	Passeriformes	Estrildidae	*Lonchura striata domestica*	SCNN1D	OWK53309.1
Aves	Passeriformes	Eurylaimidae	*Calyptomena viridis*	SCNN1D	NWI59237.1
Aves	Passeriformes	Formicariidae	*Formicarius rufipectus*	SCNN1D	NXK92902.1
Aves	Passeriformes	Fringillidae	*Loxia curvirostra*	SCNN1D	NWY94550.1
Aves	Passeriformes	Furnariidae	*Furnarius figulus*	SCNN1D	NWR85171.1
Aves	Passeriformes	Furnariidae	*Campylorhamphus procurvoides*	SCNN1D	NXC28707.1
Aves	Passeriformes	Furnariidae	*Sclerurus mexicanus*	SCNN1D	NXF80337.1
Aves	Passeriformes	Furnariidae	*Xiphorhynchus elegans*	SCNN1D	NXU88294.1
Aves	Passeriformes	Furnariidae	*Onychostruthus taczanowskii*	SCNN1D	XP_041275781.1
Aves	Passeriformes	Hirundinidae	*Hirundo rustica*	SCNN1D	NXW74284.1
Aves	Passeriformes	Hirundinidae	*Hirundo rustica rustica*	SCNN1D	RMB91608.1
Aves	Passeriformes	Icteridae	*Molothrus ater*	SCNN1D	XP_036260685.1

Class	Order	Family	Species Name	ENaC subunit	Accession
Aves	Passeriformes	Irenidae	*Chloropsis hardwickii*	SCNN1D	NWH41556.1
Aves	Passeriformes	Irenidae	*Chloropsis cyanopogon*	SCNN1D	NXP65661.1
Aves	Passeriformes	Laniidae	*Lanius ludovicianus*	SCNN1D	NWT90449.1
Aves	Passeriformes	Machaerirhynchidae	*Machaerirhynchus nigripectus*	SCNN1D	NWV99658.1
Aves	Passeriformes	Malaconotidae	*Dryoscopus gambensis*	SCNN1D	NWI81927.1
Aves	Passeriformes	Maluridae	*Daphoenositta chrysoptera*	SCNN1D	NWV52805.1
Aves	Passeriformes	Melanocharitidae	*Oreocharis arfaki*	SCNN1D	NWW10308.1
Aves	Passeriformes	Melanocharitidae	*Chaetorhynchus papuensis*	SCNN1D	NXE01244.1
Aves	Passeriformes	Melanocharitidae	*Ptilorrhoa leucosticta*	SCNN1D	NXE42273.1
Aves	Passeriformes	Menuridae	*Menura novaehollandiae*	SCNN1D	NXE94837.1
Aves	Passeriformes	Mohouidae	*Mohoua ochrocephala*	SCNN1D	NXA59613.1
Aves	Passeriformes	Monarchidae	*Ifrita kowaldi*	SCNN1D	NWW65201.1
Aves	Passeriformes	Monarchidae	*Myiagra hebetior*	SCNN1D	NXH33867.1
Aves	Passeriformes	Oriolidae	*Oriolus oriolus*	SCNN1D	NXO12356.1
Aves	Passeriformes	Passerellidae	*Melospiza melodia maxima*	SCNN1D	KAF2985723.1
Aves	Passeriformes	Passerellidae	*Melospiza melodia*	SCNN1D	NWQ58280.1
Aves	Passeriformes	Passeridae	*Passer montanus*	SCNN1D	XP_039583486.1
Aves	Passeriformes	Petroicidae	*Drymodes brunneopygia*	SCNN1D	NXU45387.1
Aves	Passeriformes	Picathartidae	*Picathartes gymnocephalus*	SCNN1D	NWI48106.1
Aves	Passeriformes	Pipridae	*Manacus vitellinus*	SCNN1D	XP_008923882.2
Aves	Passeriformes	Pipridae	*Lepidothrix coronata*	SCNN1D	XP_017681185.1
Aves	Passeriformes	Pipridae	*Pipra filicauda*	SCNN1D	XP_027584457.1
Aves	Passeriformes	Pipridae	*Chiroxiphia lanceolata*	SCNN1D	XP_032564981.1
Aves	Passeriformes	Pipritidae	*Piprites chloris*	SCNN1D	NXK36217.1
Aves	Passeriformes	Pittidae	*Pitta sordida*	SCNN1D	NWI84702.1
Aves	Passeriformes	Regulidae	*Regulus satrapa*	SCNN1D	NWR41742.1
Aves	Passeriformes	Rhagologidae	*Rhagologus leucostigma*	SCNN1D	NXB21694.1
Aves	Passeriformes	Rhinocryptidae	*Oxyruncus cristatus*	SCNN1D	NXM32830.1
Aves	Passeriformes	Rhinocryptidae	*Scytalopus superciliaris*	SCNN1D	NXP27102.1
Aves	Passeriformes	Sapayoaidae	*Sapayoa aenigma*	SCNN1D	NXA02983.1
Aves	Passeriformes	Sittidae	*Tichodroma muraria*	SCNN1D	NWH98596.1
Aves	Passeriformes	Thamnophilidae	*Chunga burmeisteri*	SCNN1D	NWS54375.1
Aves	Passeriformes	Thraupidae	*Nesospiza acunhae*	SCNN1D	NXA02619.1
Aves	Passeriformes	Thraupidae	*Camarhynchus parvulus*	SCNN1D	XP_030819914.1
Aves	Passeriformes	Tityridae	*Pachyramphus minor*	SCNN1D	NWS17542.1
Aves	Passeriformes	Tityridae	*Onychorhynchus coronatus*	SCNN1D	NWU79396.1
Aves	Passeriformes	Turdidae	*Catharus ustulatus*	SCNN1D	XP_032935794.1
Aves	Passeriformes	Tyrannidae	*Neopipo cinnamomea*	SCNN1D	NWQ59139.1
Aves	Passeriformes	Tyrannidae	*Tachuris rubrigastra*	SCNN1D	NWR29693.1

Class	Order	Family	Species Name	ENaC subunit	Accession
Aves	Passeriformes	Tyrannidae	*Smithornis capensis*	SCNN1D	NXF09216.1
Aves	Passeriformes	Tyrannidae	*Tyrannus savana*	SCNN1D	NXM03285.1
Aves	Passeriformes	Tyrannidae	*Empidonax traillii*	SCNN1D	XP_027764760.1
Aves	Passeriformes	Vangidae	*Eulacestoma nigropectus*	SCNN1D	NXB36200.1
Aves	Passeriformes	Vireonidae	*Erpornis zantholeuca*	SCNN1D	NXS82496.1
Mammalia	Monotremata	Ornithorhynchidae	*Ornithorhynchus anatinus*	SCNN1D	XP_028921777.1
Mammalia	Monotremata	Tachyglossidae	*Tachyglossus aculeatus*	SCNN1D	XP_038603243.1
Mammalia	Didelphimorphia	Microbiotheriidae	*Dromiciops gliroides*	SCNN1D	XP_043849802.1
Mammalia	Dasyuromorphia	Dasyuridae	*Sarcophilus harrisii*	SCNN1D	XP_031818927.1
Mammalia	Diprotodontia	Phascolarctidae	*Phascolarctos cinereus*	SCNN1D	XP_020827532.1
Mammalia	Diprotodontia	Phalangeridae	*Trichosurus vulpecula*	SCNN1D	XP_036599541.1
Mammalia	Diprotodontia	Vombatidae	*Vombatus ursinus*	SCNN1D	XP_027711200.1
Mammalia	Cingulata	Dasypodidae	*Dasypus novemcinctus*	SCNN1D	XP_012376989.2
Mammalia	Sirenia	Trichechidae	*Trichechus manatus latirostris*	SCNN1D	XP_023594556.1
Mammalia	Tubulidentata	Orycteropodidae	*Orycteropus afer afer*	SCNN1D	XP_042637531.1
Mammalia	Macroscelidea	Macroscelididae	*Elephantulus edwardii*	SCNN1D	XP_006886008.1
Mammalia	Afrosoricida	Chrysochloridae	*Chrysochloris asiatica*	SCNN1D	XP_006878457.1
Mammalia	Eulipotyphla	Erinaceidae	*Erinaceus europaeus*	SCNN1D	XP_007525801.1
Mammalia	Eulipotyphla	Soricidae	*Sorex araneus*	SCNN1D	XP_004607225.1
Mammalia	Chiroptera	Hipposideridae	*Hipposideros armiger*	SCNN1D	XP_019514039.1
Mammalia	Chiroptera	Molossidae	*Molossus molossus*	SCNN1D	KAF6394943.1
Mammalia	Chiroptera	Phyllostomidae	*Desmodus rotundus*	SCNN1D	XP_024410648.1
Mammalia	Chiroptera	Phyllostomidae	*Phyllostomus discolor*	SCNN1D	XP_035880991.1
Mammalia	Chiroptera	Phyllostomidae	*Artibeus jamaicensis*	SCNN1D	XP_037002859.1
Mammalia	Chiroptera	Pteropodidae	*Pteropus vampyrus*	SCNN1D	XP_011375630.1
Mammalia	Chiroptera	Pteropodidae	*Rousettus aegyptiacus*	SCNN1D	XP_036079706.1
Mammalia	Chiroptera	Pteropodidae	*Pteropus giganteus*	SCNN1D	XP_039732735.1
Mammalia	Chiroptera	Vespertilionidae	*Myotis davidii*	SCNN1D	ELK23616.1
Mammalia	Chiroptera	Vespertilionidae	*Eptesicus fuscus*	SCNN1D	XP_028016669.1
Mammalia	Chiroptera	Vespertilionidae	*Myotis myotis*	SCNN1D	XP_036163388.1
Mammalia	Chiroptera	Vespertilionidae	*Pipistrellus kuhlii*	SCNN1D	XP_036316875.1
Mammalia	Pholidota	Manidae	*Manis javanica*	SCNN1D	XP_017534879.2
Mammalia	Pholidota	Manidae	*Manis pentadactyla*	SCNN1D	XP_036738603.1
Mammalia	Carnivora	Canidae	*Nyctereutes procyonoides*	SCNN1D	CAD7689419.1
Mammalia	Carnivora	Canidae	*Vulpes vulpes*	SCNN1D	XP_025862713.1
Mammalia	Carnivora	Canidae	*Canis lupus dingo*	SCNN1D	XP_035571831.1
Mammalia	Carnivora	Canidae	*Canis lupus familiaris*	SCNN1D	XP_038522238.1
Mammalia	Carnivora	Felidae	*Lynx pardinus*	SCNN1D	VFV29629.1
Mammalia	Carnivora	Felidae	*Panthera tigris altaica*	SCNN1D	XP_015398634.1

Class	Order	Family	Species Name	ENaC subunit	Accession
Mammalia	Carnivora	Felidae	*Panthera pardus*	SCNN1D	XP_019316581.1
Mammalia	Carnivora	Felidae	*Acinonyx jubatus*	SCNN1D	XP_026916564.1
Mammalia	Carnivora	Felidae	*Lynx canadensis*	SCNN1D	XP_032450062.1
Mammalia	Carnivora	Felidae	*Puma yagouaroundi*	SCNN1D	XP_040314080.1
Mammalia	Carnivora	Herpestidae	*Suricata suricatta*	SCNN1D	XP_029801812.1
Mammalia	Carnivora	Hyaenidae	*Hyaena hyaena*	SCNN1D	XP_039104244.1
Mammalia	Carnivora	Mustelidae	*Mustela putorius furo*	SCNN1D	XP_012905866.1
Mammalia	Carnivora	Mustelidae	*Lontra canadensis*	SCNN1D	XP_032719009.1
Mammalia	Carnivora	Otariidae	*Callorhinus ursinus*	SCNN1D	XP_025712406.1
Mammalia	Carnivora	Otariidae	*Zalophus californianus*	SCNN1D	XP_027470810.2
Mammalia	Carnivora	Phocidae	*Phoca vitulina*	SCNN1D	XP_032271219.1
Mammalia	Carnivora	Phocidae	*Halichoerus grypus*	SCNN1D	XP_035945638.1
Mammalia	Carnivora	Phocidae	*Neomonachus schauinslandi*	SCNN1D	XP_021540026.1
Mammalia	Carnivora	Phocidae	*Mirounga leonina*	SCNN1D	KAF3820439.1
Mammalia	Carnivora	Ursidae	*Ailuropoda melanoleuca*	SCNN1D	XP_034526906.1
Mammalia	Perissodactyla	Equidae	*Equus przewalskii*	SCNN1D	XP_008529865.1
Mammalia	Perissodactyla	Equidae	*Equus asinus*	SCNN1D	XP_014718579.1
Mammalia	Perissodactyla	Rhinocerotidae	*Diceros bicornis minor*	SCNN1D	KAF5927193.1
Mammalia	Perissodactyla	Rhinocerotidae	*Ceratotherium simum simum*	SCNN1D	XP_014639206.1
Mammalia	Artiodactyla	Bovidae	*Bos taurus*	SCNN1D	XP_005217258.1
Mammalia	Artiodactyla	Bovidae	*Bos mutus*	SCNN1D	XP_005888969.1
Mammalia	Artiodactyla	Bovidae	*Bubalus bubalis*	SCNN1D	XP_006067018.1
Mammalia	Artiodactyla	Bovidae	*Bison bison bison*	SCNN1D	XP_010848530.1
Mammalia	Artiodactyla	Bovidae	*Ovis aries*	SCNN1D	XP_027831921.1
Mammalia	Artiodactyla	Bovidae	*Oryx dammah*	SCNN1D	XP_040120096.1
Mammalia	Artiodactyla	Camelidae	*Camelus bactrianus*	SCNN1D	XP_010956130.1
Mammalia	Artiodactyla	Camelidae	*Camelus dromedarius*	SCNN1D	XP_031319641.1
Mammalia	Artiodactyla	Camelidae	*Vicugna pacos*	SCNN1D	XP_031529322.1
Mammalia	Artiodactyla	Camelidae	*Camelus ferus*	SCNN1D	XP_032350794.1
Mammalia	Artiodactyla	Cervidae	*Muntiacus muntjak*	SCNN1D	KAB0341096.1
Mammalia	Artiodactyla	Cervidae	*Muntiacus reevesi*	SCNN1D	KAB0354034.1
Mammalia	Artiodactyla	Cervidae	*Cervus hanglu yarkandensis*	SCNN1D	KAF4018533.1
Mammalia	Artiodactyla	Cervidae	*Cervus elaphus hippelaphus*	SCNN1D	OWK08439.1
Mammalia	Artiodactyla	Cervidae	*Odocoileus virginianus texanus*	SCNN1D	XP_020729446.1
Mammalia	Artiodactyla	Suidae	*Sus scrofa*	SCNN1D	XP_020953090.1
Mammalia	Scandentia	Tupaiidae	*Tupaia chinensis*	SCNN1D	XP_027622342.1
Mammalia	Rodentia	Bathyergidae	*Heterocephalus glaber*	SCNN1D	XP_004863993.1
Mammalia	Rodentia	Bathyergidae	*Fukomys damarensis*	SCNN1D	XP_010625992.1
Mammalia	Rodentia	Castoridae	*Castor canadensis*	SCNN1D	XP_020011157.1

Class	Order	Family	Species Name	ENaC subunit	Accession
Mammalia	Rodentia	Caviidae	*Cavia porcellus*	SCNN1D	XP_003461599.2
Mammalia	Rodentia	Chinchillidae	*Chinchilla lanigera*	SCNN1D	XP_005404383.1
Mammalia	Rodentia	Dipodidae	*Jaculus jaculus*	SCNN1D	XP_004657476.1
Mammalia	Rodentia	Echimyidae	*Echinops telfairi*	SCNN1D	XP_030743899.1
Mammalia	Rodentia	Octodontidae	*Octodon degus*	SCNN1D	XP_004639733.1
Mammalia	Rodentia	Spalacidae	*Nannospalax galili*	SCNN1D	XP_008850903.2
Mammalia	Dermoptera	Cynocephalidae	*Galeopterus variegatus*	SCNN1D	XP_008576389.1
Mammalia	Primates	Aotidae	*Aotus nancymaae*	SCNN1D	XP_021523120.1
Mammalia	Primates	Cebidae	*Saimiri boliviensis boliviensis*	SCNN1D	XP_010348668.2
Mammalia	Primates	Cebidae	*Sapajus apella*	SCNN1D	XP_032117895.1
Mammalia	Primates	Cebidae	*Callithrix jacchus*	SCNN1D	XP_035108548.1
Mammalia	Primates	Cebidae	*Cebus imitator*	SCNN1D	XP_037599404.1
Mammalia	Primates	Cercopithecidae	*Rhinopithecus bieti*	SCNN1D	XP_017750698.1
Mammalia	Primates	Cercopithecidae	*Carlito syrichta*	SCNN1D	XP_021563524.1
Mammalia	Primates	Cercopithecidae	*Rhinopithecus roxellana*	SCNN1D	XP_010375489.1
Mammalia	Primates	Cercopithecidae	*Macaca nemestrina*	SCNN1D	XP_011768647.2
Mammalia	Primates	Cercopithecidae	*Colobus angolensis palliatus*	SCNN1D	XP_011811772.1
Mammalia	Primates	Cercopithecidae	*Mandrillus leucophaeus*	SCNN1D	XP_011857366.1
Mammalia	Primates	Cercopithecidae	*Cercocebus atys*	SCNN1D	XP_011886575.1
Mammalia	Primates	Cercopithecidae	*Theropithecus gelada*	SCNN1D	XP_025242263.1
Mammalia	Primates	Cercopithecidae	*Macaca mulatta*	SCNN1D	XP_028702982.1
Mammalia	Primates	Cercopithecidae	*Papio anubis*	SCNN1D	XP_031519213.1
Mammalia	Primates	Cercopithecidae	*Piliocolobus tephrosceles*	SCNN1D	XP_031790725.1
Mammalia	Primates	Cercopithecidae	*Trachypithecus francoisi*	SCNN1D	XP_033084661.1
Mammalia	Primates	Cercopithecidae	*Chlorocebus sabaeus*	SCNN1D	XP_037841154.1
Mammalia	Primates	Hominidae	*Homo sapiens*	SCNN1D	AAI25075.1
Mammalia	Primates	Hominidae	*Pan troglodytes*	SCNN1D	NP_001009072.1
Mammalia	Primates	Hominidae	*Pongo abelii*	SCNN1D	PNJ04390.1
Mammalia	Primates	Hominidae	*Gorilla gorilla gorilla*	SCNN1D	XP_004024514.3
Mammalia	Primates	Hylobatidae	*Nomascus leucogenys*	SCNN1D	XP_030661122.1
Mammalia	Primates	Hylobatidae	*Hylobates moloch*	SCNN1D	XP_032614469.1
