# Supplementary material for: Varying Selection Pressure for a Na+ Sensing Site in Epithelial Na+ Channel Subunits Reflect Divergent Roles in Na+ Homeostasis
Source: Mol Biol Evol. 2024 Aug 5;41(8):msae162. doi: 10.1093/molbev/msae162 (PMC11331422; doi:10.1093/molbev/msae162)
Supplement: msae162_Supplementary_Data [file msae162_supplementary_data.zip › Supplementary Table 2.pdf]

Supplementary Table 2. Substitution of Asp critical for Na^+ self-inhibition (aligning with human Asp-338) in SCNN1A and SCNN1D from various species.

Class / Order / Family	Species name	Asp substitution
	**SCNN1A**	
Myxini		
Myxiniformes		
Myxinidae	*Eptatretus burgeri*	Asn
Mammalia		
Pilosa		
Choloepodidae	*Choloepus didactylus*	Gly
	**SCNN1D**	
Amphibia		
Gymnophiona		
Siphonopidae	*Microcaecilia unicolor*	Asn
Dermophiidae	*Geotrypetes seraphini*	Asn
Mammalia		
Monotremata		
Ornithorhynchidae	*Ornithorhynchus anatinus*	Lys
Tachyglossidae	*Tachyglossus aculeatus*	Asn
Mammalia:Marsupialia		
Dasyuromorphia		
Dasyuridae	*Sarcophilus harrisii*	Ser
Diprotodontia		
Phalangeridae	*Trichosurus vulpecula*	His
Microbiotheria		
Microbiotheriidae	*Dromiciops gliroides*	Asn
Mammalia		
Rodentia		
Caviidae	*Cavia porcellus*	Val
Octodontidae	*Octodon degus*	Val
Heterocephalidae	*Heterocephalus glaber*	Val
Chinchillidae	*Chinchilla lanigera*	Val
Bathyergidae	*Fukomys damarensis*	Val
Castoridae	*Castor canadensis*	Tyr
Cingulata		
Dasypodidae	*Dasypus novemcinctus*	Gly
Primates		
Aotidae	*Aotus nancymaae*	Ala
Tarsiidae	*Carlito syrichta*	Ala
Callitrichidae	*Callithrix jacchus*	Ala
Cebidae	*Cebus imitator*	Ala

Cebidae	*Sapajus apella*	Ala
Cebidae	*Saimiri boliviensis boliviensis*	Ala
Hominidae	*Pan troglodytes*	Pro
Hominidae	*Pongo abelii*	Pro
Hominidae	*Gorilla gorilla gorilla*	Pro
Hominidae	*Homo sapiens*	Pro
Cercopithecidae	*Rhinopithecus roxellana*	Pro
Cercopithecidae	*Macaca nemestrina*	Pro
Cercopithecidae	*Colobus angolensis palliatus*	Pro
Cercopithecidae	*Mandrillus leucophaeus*	Pro
Cercopithecidae	*Cercocebus atys*	Pro
Cercopithecidae	*Theropithecus gelada*	Pro
Cercopithecidae	*Macaca mulatta*	Pro
Cercopithecidae	*Papio anubis*	Pro
Cercopithecidae	*Piliocolobus tephrosceles*	Pro
Cercopithecidae	*Trachypithecus francoisi*	Pro
Cercopithecidae	*Chlorocebus sabaeus*	Pro
Hylobatidae	*Hylobates moloch*	Pro
Hylobatidae	*Nomascus leucogenys*	Pro
Eulipotyhia		
Erinaceidae	*Erinaceus europaeus*	Lys
Soricidae	*Sorex araneus*	Pro
Carnivora		
Mustelidae	*Mustela putorius furo*	His
Mustelidae	*Lontra canadensis*	His
Phocidae	*Neomonachus schauinslandi*	Asn
Phocidae	*Mirounga leonina*	Pro
Aves		
Passeriformes		
Tichodromidae	*Tichodroma muraria*	Ala
Turdidae	*Catharus ustulatus*	Ala
Chloropseidae	*Chloropsis hardwickii*	Glu
Chloropseidae	*Chloropsis cyanopogon*	Glu
Estrildidae	*Lonchura striata domestica*	Glu
Urocynchramidae	*Urocynchramus pylzowi*	Glu
Passeridae	*Passer montanus*	Glu
Passeridae	*Onychostruthus taczanowskii*	Glu
Cardinalidae	*Cardinalis cardinalis*	Glu
Icteridae	*Molothrus ater*	Glu
Fringillidae	*Loxia curvirostra*	Glu
Cardinalidae	*Pheucticus melanocephalus*	Glu

Passerellidae	*Melospiza melodia maxima*	Glu
Passerellidae	*Melospiza melodia*	Glu
Thraupidae	*Nesospiza acunhae*	Glu
Thraupidae	*Camarhynchus parvulus*	Glu
