# Supplementary material for: Varying Selection Pressure for a Na+ Sensing Site in Epithelial Na+ Channel Subunits Reflect Divergent Roles in Na+ Homeostasis
Source: Mol Biol Evol. 2024 Aug 5;41(8):msae162. doi: 10.1093/molbev/msae162 (PMC11331422; doi:10.1093/molbev/msae162)
Supplement: msae162_Supplementary_Data [file msae162_supplementary_data.zip › Supplementary Table 3.pdf]

Primers for RT-PCR and expected product sizes.

Target		Primer sequence (5'–3')	Amplicon (bp)
Tupaia belangeri			
ENaC α	forward	AACGACTGACGACCAACCAA	373
	reverse	GAAGCTTCTAGGCTGCGGAA	
ENaC β	forward	ATTGCTACTCGGATCTGCGG	206
	reverse	GACAACTCCTTTCCGGCTCA	
ENaC γ	forward	GCCCAGCCAACAGTATCGAG	260
	reverse	GAAAGTGGGTGGGTCATCGT	
ENaC δ	forward	ACCTTGGGCATGCTCTACTG	802
	reverse	GGTGCACCTCATCCTCACGA	
GAPDH	forward	GCCTGGAGAAAGCTGCCAAAT	378
	reverse	ACTGTCAAGGAGGGGAGCTT	
Gallus gallus			
ENaC α	forward	CCGCGGTGGTTCTGTGAAG	939
	reverse	ACCACCAGAGAGAGGCCATT	
ENaC β	forward	ACTGGGGCATGGACAAAGAG	340
	reverse	AAGGGAAGAGGGAGCTGTATG	
ENaC γ	forward	GTGACATCGACAGGAGCCAA	621
	reverse	CAGAAGGATGACGAGCGTGT	
ENaC δ	forward	GAGCCAGGGATACCATCTGC	387
	reverse	TGTGTCGCTGCTCTTGTCAT	
GAPDH	forward	CACTATCTTCCAGGAGCGTGA	540
	reverse	TTGGCTGGTTTCTCCAGACG	
